# Supplementary material for: Molecular Analysis of the Official Algerian Olive Collection Highlighted a Hotspot of Biodiversity in the Central Mediterranean Basin
Source: Genes (Basel). 2020 Mar 13;11(3):303. doi: 10.3390/genes11030303 (PMC7140851; doi:10.3390/genes11030303)
Supplement: Supplementary file 1 [file genes-11-00303-s001.zip › 16_Table_S6.docx]

**Article Title: Molecular analysis of the official Algerian olive collection highlighted a hotspot of biodiversity in the Central Mediterranean basin**

**Journal:** Genes

**Authors:** Haddad Benalia, Alessandro Silvestre Gristina, Francesco Mercati, Saadi Abd Elkader, Haddad Nassima, Adriana Martorana, Abdoallah Sharaf, Francesco Carimi.

**Correspondence:** alessandro.gristina@ibbr.cnr.it

**Table S6.** Genetic parameters at 16 nuSSR and 6 cpSSR loci used to genotype the ITAFV Algerian germplasm collection

| **Marker** | **Type** | **Size range** | **Major allele frequency** | **Na** | **Ne** | **Ho** | **He** | **F** | **PIC** |
| --- | --- | --- | --- | --- | --- | --- | --- | --- | --- |
| DCA07 | nuSSR | 121-165 | 0.544 | 11 | 2.883 | 0.294 | 0.653 | 0.550 | 0.622 |
| DCA09 | nuSSR | 150-206 | 0.382 | 10 | 4.007 | 0.882 | 0.750 | -0.176 | 0.715 |
| DCA11 | nuSSR | 130-160 | 0.297 | 7 | 4.385 | 0.375 | 0.772 | 0.514 | 0.765 |
| DCA13 | nuSSR | 120-140 | 0.338 | 6 | 3.584 | 0.765 | 0.721 | -0.061 | 0.670 |
| DCA15 | nuSSR | 247-269 | 0.529 | 6 | 2.563 | 0.471 | 0.610 | 0.228 | 0.545 |
| DCA16 | nuSSR | 122-178 | 0.471 | 8 | 3.607 | 0.735 | 0.723 | -0.017 | 0.696 |
| DCA18 | nuSSR | 168-184 | 0.294 | 8 | 4.857 | 0.706 | 0.794 | 0.111 | 0.765 |
| EMO90 | nuSSR | 183-195 | 0.529 | 5 | 2.796 | 0.882 | 0.642 | -0.374 | 0.596 |
| GAPU47 | nuSSR | 164-184 | 0.588 | 5 | 2.473 | 0.824 | 0.596 | -0.383 | 0.553 |
| GAPU59 | nuSSR | 208-224 | 0.516 | 4 | 2.817 | 0.500 | 0.645 | 0.225 | 0.639 |
| GAPU71A | nuSSR | 188-242 | 0.706 | 7 | 1.897 | 0.500 | 0.473 | -0.058 | 0.443 |
| GAPU71B | nuSSR | 120-146 | 0.25 | 8 | 5.161 | 1.000 | 0.806 | -0.240 | 0.778 |
| GAPU101 | nuSSR | 184-218 | 0.206 | 9 | 6.568 | 1.000 | 0.848 | -0.180 | 0.829 |
| GAPU103 | nuSSR | 137-187 | 0.338 | 9 | 4.919 | 0.941 | 0.797 | -0.181 | 0.771 |
| UDO01 | nuSSR | 137-145 | 0.359 | 5 | 3.282 | 0.688 | 0.695 | 0.011 | 0.678 |
| UDO43 | nuSSR | 171-221 | 0.697 | 7 | 1.914 | 0.364 | 0.478 | 0.238 | 0.474 |
| ***Mean*** |  |  |  | *7.2* | *3.6* | *0.7* | *0.7* | *-* | *0.659* |
|  |  |  |  |  |  |  |  |  |  |
|  |  |  |  |  |  |  |  |  |  |
| **ccmp5** | cpSSR | 102-108 | 0.647 | 3 |  |  |  |  |  |
| **ccmp7** | cpSSR | 119-121 | 0.794 | 2 |  |  |  |  |  |
| **psbK-trnS-polyT/A** | cpSSR | 109-111 | 0.892 | 3 |  | - | - |  | - |
| **trnG-polyT** | cpSSR | 106-117 | 0.875 | 3 |  | - | - |  | - |
| **trnS-G-indel1** | cpSSR | 81-82 | 0.982 | 2 |  | - | - |  | - |
| **trnT-L-polyT** | cpSSR | 87 | 1.000 | 1 |  | - | - |  | - |
| ***Mean*** |  |  | *0.865* | *2.3* |  |  |  |  |  |

***Na*** *= Number of allele;* ***Ne****= Number of effective alleles* ***Ho*** *= Observed heterozygosity;* ***He*** *= Expected heterozygosity;* ***F****= Fixation index;* ***PIC*** *= Polymorphic Information Content.*
