# Supplementary material for: Molecular Analysis of the Official Algerian Olive Collection Highlighted a Hotspot of Biodiversity in the Central Mediterranean Basin
Source: Genes (Basel). 2020 Mar 13;11(3):303. doi: 10.3390/genes11030303 (PMC7140851; doi:10.3390/genes11030303)
Supplement: Supplementary file 1 [file genes-11-00303-s001.zip › 11_Table_S1.pdf]

Table S1 Name, synonym, Arabic name, meaning, geographic diffusion and origin, use of fruits and oil percentage of analysed Algerian varieties

Cultivar Name	Synonyms	Arab cultivar name	Meaning	Diffusion at national level	Origin	Localisation	Utilisation	Oil %
**Abani**	Laabani	عباني	Name of the region of provenance	Limited	Valley Oued El Arab Cherchar (Khenchela)	Northeast	Olive oil	16-20%
**Aberkane**	Averkane	أبركان	Black fruit	Limited	Akbou (Bejaia)	Northeast	Double use	16-20%
**Aeleh**	Aaleh	أيلاح	Name of the region of provenance	Limited	Cherchar (Khenchela)	Northeast	Olive oil	18-20%
**Aghchren d'el Ousseur**	-	أغشن دالعصور	Name of the region of provenance	Limited	Bougaa (Sétif)	Northeast	Double use	16-20%
**Aghchren de Titest**	-	أغشن تيتست	Name of the region of provenance	Limited	Hammam Guergour (Sétif)	Northeast	Double use	14-18%
**Aghenfas**	Aghenfous	أغنفاس	Nose-shaped fruit	Limited	Bougaa (Sétif)	Northeast	Double use	16-20%
**Agrarez**	-	أقرارز	Rounded-shaped fruit	Limited	Tazmalt (Bejaia)	Northeast	Double use	16-20%
**Aguenaou**	Agnaw	أقناون	Dark-black fruit	Limited	Bousselah (Sétif)	Northeast	Double use	16-20%
**Aimel**	Ayemel, Haimel	أيمال	Name of the region of provenance	Limited	Ait Aimel (Bejaia)	Northeast	Olive oil	18-22%
**Akerma**	-	أكرمة	Name of the region of provenance	Limited	Hammam Guergour (Sétif)	Northeast	Double use	18-22%
**Azeradj**	Aradj, Adjeraz	أزراج	Piquant fruit	Ten percent of the national olive orchard	Sedouk (Bejaia)	Northeast	Double use	24-28%
**Blanquette de Guelma**	-	بلانكات قالمة	White fruit from Guelma	Widespread in the north-east of Constantine	Guelma	Northeast	Olive oil	18-22%
**Bouchouk Guergour**	-	بو شوك قرقور	Thorn fruit from Guergour	Limited	Guergour (Sétif)	Northeast	Double use	22-26%
**Bouchouk Lafayette**	-	بو شوك لافاييت	Thorn fruit from Lafayette	Limited	Bougaa (Sétif)	Northeast	Double use	22-26%
**Bouchouk Soummam**	Bouchouk Sidi Aich, Avouchouk	بوشوك صومام	Thorn fruit from Soummam	Vallée d'Oued Soummam	Valley d'Oued Soummam, Sidi Aich (Bejaia)	Northeast	Double use	22-26%
**Boughenfous**	-	بوغنفوس	-	Limited	Bouandas (Setif)	Northeast	Olive oil	22-26%
**Bouichret**	Boutichrat, Avouichert	بويشرط	Berber tattoo	Local	Tazmalt (Bejaia)	Northeast	Olive oil	20-24%
**Boukaila**	-	بوكيلة	Name of the region of provenance	Limited	Constantine	Northeast	Olive oil	16-20%
**Bouricha**	Bouricha, Olive d'el Arrouch	بوريشة	Pen tip tab	Limited	Elharouch (Skikda)	Northeast	Olive oil	18-22%
**Chemlal**	Achamlal, Achamli, Achemlal	شملال	White fruit	Forty percent of the national olive orchard	Kabylie	North central and Northeast	Olive oil	18-22%
**Ferkani**	Ferkane	فركاني	Name of the region of provenance	Aurès region	Ferkane (Tébessa)	Northeast	Olive oil	28-32%
**Grosse du Hamma**	La Grosse de Hamma, Qelb Ehtour, Coeur de bœuf	كبيرة الحامة	Name of the region of provenance	Limited	Constantine	Northeast	Double use	16-20%
**Hamra**	Rougette, Roussette	حمراء	Red fruit	Widespread in the north of Constantine	Jijel	Northeast	Olive oil	18-22%
**Limli**	Imeli, Limeli	ليملي	Name of the region of provenance	Eight percent of the national olive orchard	Sidi Aich (Bejaia)	Northeast	Olive oil	20-24%
**Longue de Miliana**	-	طويلة مليانة	Long fruit from Miliana	Local	Khemis Miliana (Ain Defla)	North central and Northwest	Double use	16-20%
**Mekki**	-	مكي	Name of the region of provenance	Limited	Khenchela	Northeast	Olive oil	12-16%
**Neb Djemel**	-	ناب الجمل	Camel tooth	Limited	Valley D'Oued El Arab Cherchar (Khenchela)	Northeast	Olive oil	16-20%
**Ronde de Miliana**	-	مستديرة مليانة	Round fruit from Miliana	Limited	Valley Miliana (Ain Defla)	North central and Northwest	Double use	16-20%
**Rougette de Mitidja**	-	حمراء متيجة	Red fruit from Mitidja	Limited	Plain de Mitidja (Blida)	North central	Olive oil	18-20%
**Sigoise**	Olive de Tlemcen, Olive du Tell	سجواز	Name of the region of provenance	Twenty-five of the national olive orchard	Plain of Sig (Mascara)	Northwest	Double use	18-22%
**Souidi**	-	سويدي	Dark-black fruit	Local	Cherchar (Khenchela)	Northeast	Olive oil	-
**Tabelout**	Tabelout, Abelout	تبلوط	Oak's fruit	Limited	Mountainous area of Bejaia golf, Nord side of Babors (Bejaia)	Northeast	Olive oil	20-24%
**Takesrit**	-	تكسريت	Name of the region of provenance	Local	El Kseur (Bejaia)	Northeast	Olive oil	16-20%
**Tefah**	Atefah, Tefahi	تفاح	Apple-shaped fruits	Limited	Sedouk (Bejaia)	Northeast	Olive oil	18-22%
