# Supplementary material for: Molecular Analysis of the Official Algerian Olive Collection Highlighted a Hotspot of Biodiversity in the Central Mediterranean Basin
Source: Genes (Basel). 2020 Mar 13;11(3):303. doi: 10.3390/genes11030303 (PMC7140851; doi:10.3390/genes11030303)

Molecular analysis of the official Algerian olive collection highlighted a hotspot of biodiversity in the Central Mediterranean basin

Journal: Genes

Authors: Haddad Benalia, Alessandro Silvestre Gristina, Francesco Mercati, Saadi Abd Elkader, Haddad Nassima, Adriana Martorana, Abdoallah Sharaf, Francesco Carimi.

Correspondence: alessandro.gristina@ibbr.cnr.it

Table S2. Catalogue of the official Algerian varieties analysed

عباني
Abani

Characters of the tree
Plant vigour: Medium
Growth habit: Erect
Canopy vegetation: Medium

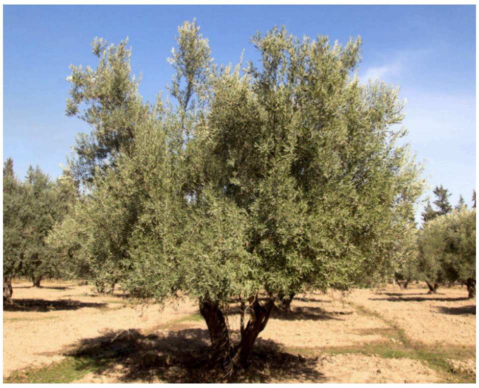

Characters of the leaf
Shape: Lanceolate
Blade length: Long
Blade width: Medium

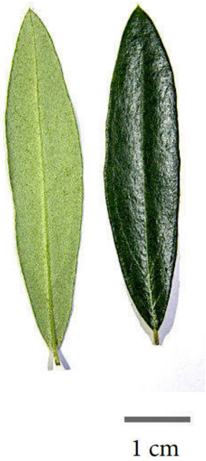

Characters of the inflorescence
Length: Medium
Number of flowers for inflorescence: Medium

Characters of the fruit
Weight: Low
Shape: Elongated
Symmetry: Asymmetric
Position of maximum transverse diameter: Towards base
Apex: Pointed
Base: Rounded
Nipple: Absent
Lenticels number: Few
Lenticels dimension: Small

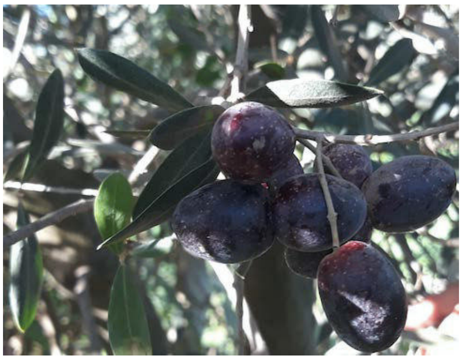

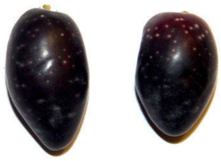

Characters of the endocarp
Weight: Medium
Shape: Elongated
Symmetry: Asymmetric
Position of maximum transverse diameter: Central apex
Apex: Pointed
Base: Rounded
Surface: Smooth
Number of grooves: Medium
Distribution of grooves: Uniform

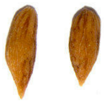

أبركان
Aberkane

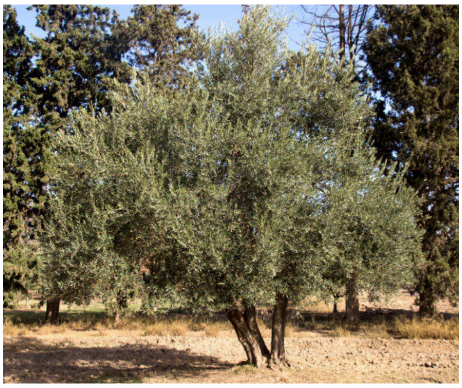

Characters of the tree
Plant vigour: Medium
Growth habit: Erect
Canopy vegetation: Medium

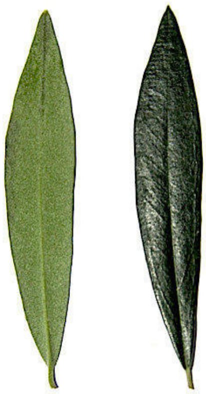

Characters of the leaf
Shape: Lanceolate
Blade length: Long
Blade width: Medium

Characters of the inflorescence
Length: Short
Number of flowers for inflorescence: Low

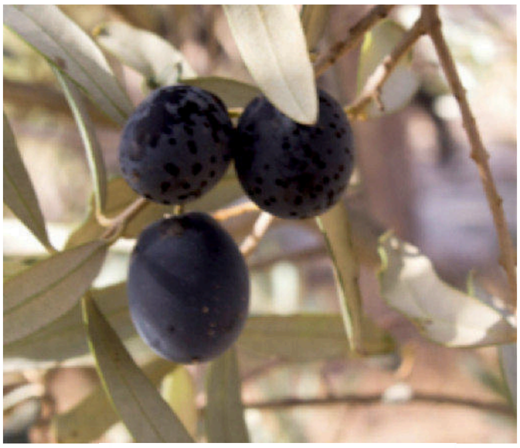

Characters of the fruit
Weight: High
Shape: Elongated
Symmetry: Slightly asymmetric
Position of maximum transverse diameter: Central
Apex: Rounded
Base: Rounded
Nipple: Absent
Lenticels number: Few
Lenticels dimension: Large

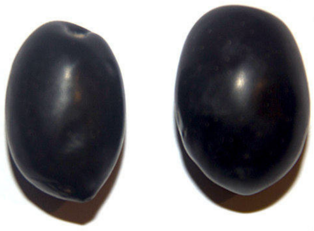

Characters of the endocarp
Weight: High
Shape: Elongated
Symmetry: Slightly asymmetric
Position of maximum transverse diameter: Towards apex
Apex: Rounded
Base: Rounded
Surface: Rugose
Number of grooves: Medium
Distribution of grooves: Uniform

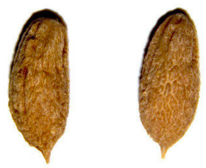

أيلاح
Aeleh

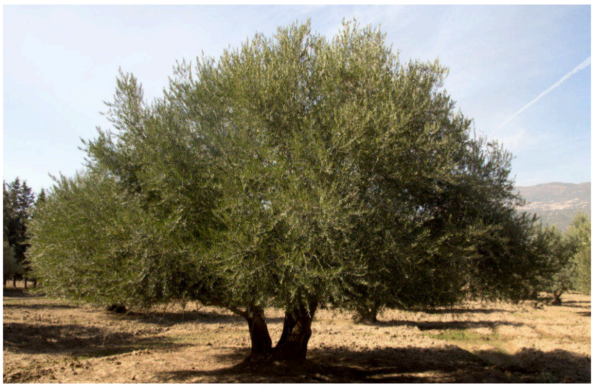

Characters of the tree
Plant vigour: Medium
Growth habit: Spreading
Canopy vegetation: Dense

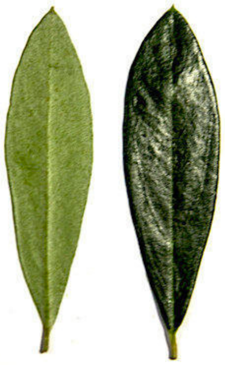

Characters of the leaf
Shape: Elliptic-Lanceolate
Blade length: Long
Blade width: Medium

Characters of the inflorescence
Length: Medium
Number of flowers for inflorescence: Medium

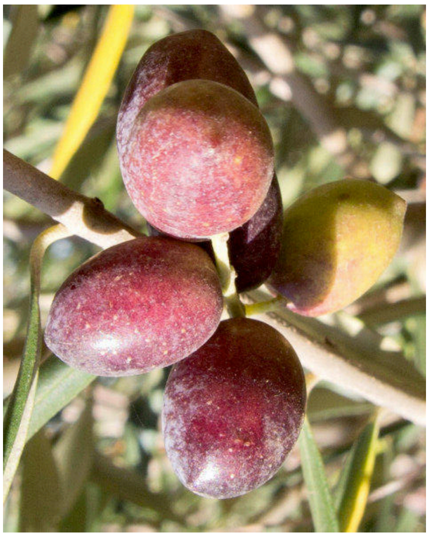

Characters of the fruit
Weight: Medium
Shape: Elongated
Symmetry: Asymmetric
Position of maximum transverse diameter: Towards base
Apex: Pointed
Base: Rounded
Nipple: Absent
Lenticels number: Few
Lenticels dimension: Small

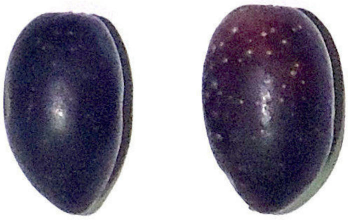

Characters of the endocarp
Weight: Medium
Shape: Elongated
Symmetry: Asymmetric
Position of maximum transverse diameter: Central apex
Apex: Pointed
Base: Rounded
Surface: Smooth
Number of grooves: High
Distribution of grooves: Uniform

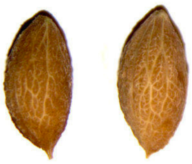

أغشن دالعصور

Aghchren d'el Ousseur

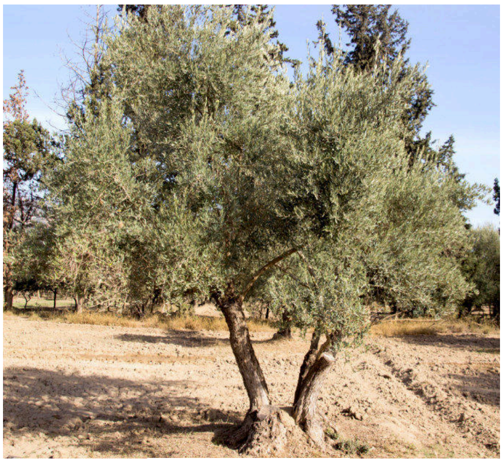

Characters of the tree
Plant vigour: Weak
Growth habit: Spreading
Canopy vegetation: Dense

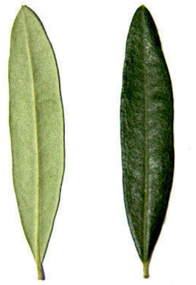

Characters of the leaf
Shape: Elliptic-Lanceolate
Blade length: Medium
Blade width: Medium

Characters of the inflorescence
Length: Short
Number of flowers for inflorescence: Low

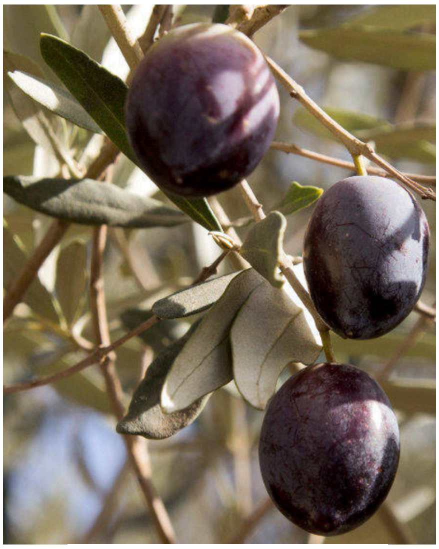

Characters of the fruit
Weight: High
Shape: Elongated
Symmetry: Asymmetric
Position of maximum transverse diameter: Central
Apex: Pointed
Base: Truncate
Nipple: Tenuous
Lenticels number: Few
Lenticels dimension: Large

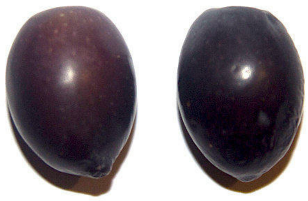

Characters of the endocarp
Weight: High
Shape: Elliptic
Symmetry: Slightly asymmetric
Position of maximum transverse diameter: Towards apex
Apex: Pointed
Base: Rounded
Surface: Rugose
Number of grooves: Medium
Distribution of grooves: Uniform

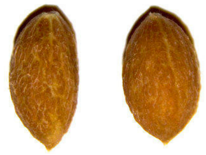

أغشن تيتست

Aghchren de Titest

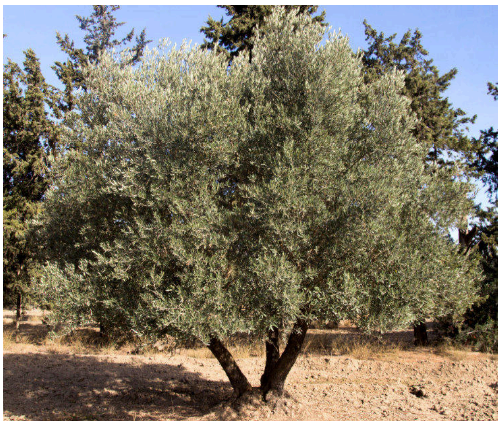

Characters of the tree
Plant vigour: Weak
Growth habit: Spreading
Canopy vegetation: Sparse

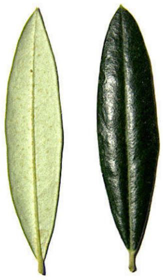

Characters of the leaf
Shape: Lanceolate
Blade length: Medium
Blade width: Narrow

Characters of the inflorescence
Length: Short
Number of flowers for inflorescence: Medium

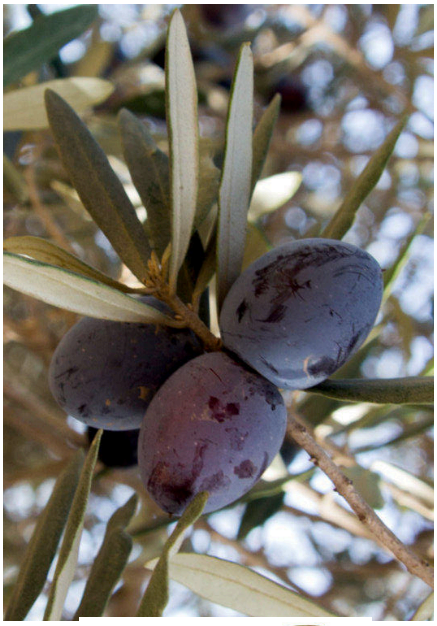

Characters of the fruit
Weight: Medium
Shape: Spherical
Symmetry: Slightly asymmetric
Position of maximum transverse diameter: Central
Apex: Rounded
Base: Rounded
Nipple: Absent
Lenticels number: Few
Lenticels dimension: Large

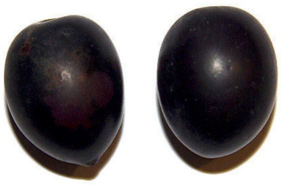

Characters of the endocarp
Weight: Low
Shape: Ovoid
Symmetry: Slightly asymmetric
Position of maximum transverse diameter: Central apex
Apex: Rounded
Base: Rounded
Surface: Rugose
Number of grooves: Medium
Distribution of grooves: Uniform

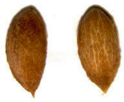

أغنفاس
Aghenfas

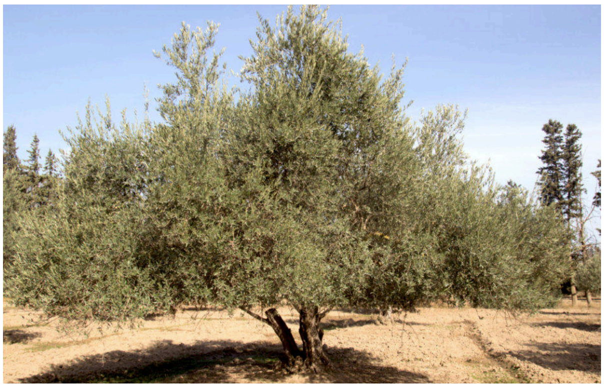

Characters of the tree
Plant vigour: Medium
Growth habit: Spreading
Canopy vegetation: Medium

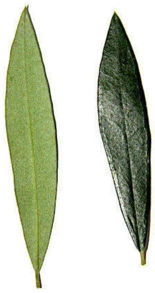

Characters of the leaf
Shape: Lanceolate
Blade length: Medium
Blade width: Medium

Characters of the inflorescence
Length: Short
Number of flowers for inflorescence: Low

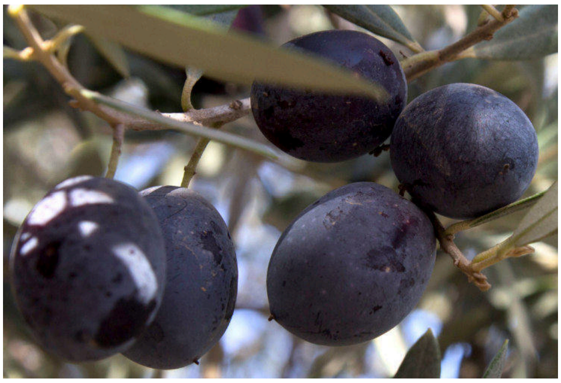

Characters of the fruit
Weight: High
Shape: Elongated
Symmetry: Slightly asymmetric
Position of maximum transverse diameter: Central
Apex: Pointed
Base: Rounded
Nipple: Tenuous
Lenticels number: Few
Lenticels dimension: Large

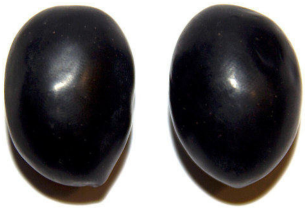

Characters of the endocarp
Weight: High
Shape: Elliptic
Symmetry: Asymmetric
Position of maximum transverse diameter: Towards apex
Apex: Rounded
Base: Pointed
Surface: Rugose
Number of grooves: Medium
Distribution of grooves: Uniform

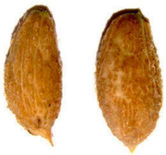

أقرارز
Agrarez

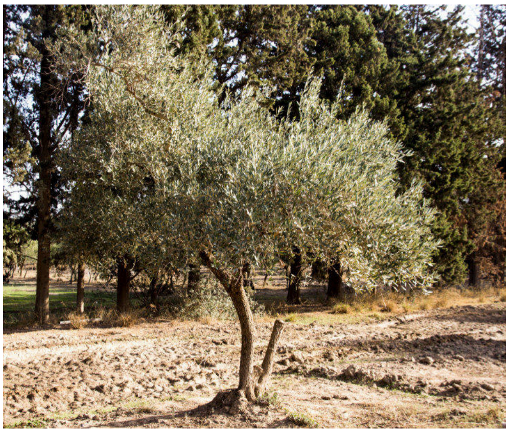

Characters of the tree
Plant vigour: Weak
Growth habit: Spreading
Canopy vegetation: Medium

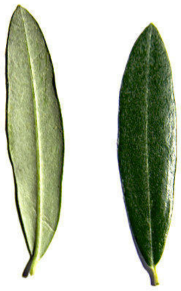

Characters of the leaf
Shape: Elliptic-Lanceolate
Blade length: Medium
Blade width: Medium

Characters of the inflorescence
Length: Short
Number of flowers for inflorescence: Low

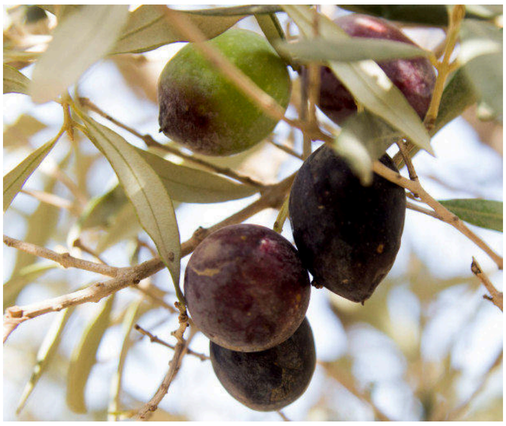

Characters of the fruit
Weight: Medium
Shape: Spherical
Symmetry: Slightly asymmetric
Position of maximum transverse diameter: Central
Apex: Pointed
Base: Rounded
Nipple: Absent
Lenticels number: Few
Lenticels dimension: Large

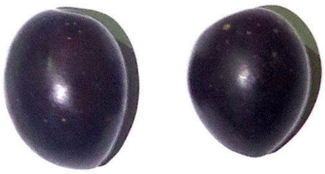

Characters of the endocarp
Weight: Medium
Shape: Ovoid
Symmetry: Slightly asymmetric
Position of maximum transverse diameter: Central apex
Apex: Rounded
Base: Rounded)
Surface: Rugose
Number of grooves: Medium
Distribution of grooves: Uniform

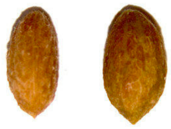

أقنا ون
Aguenaou

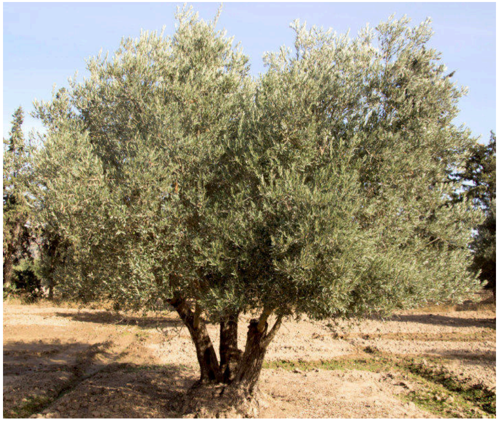

Characters of the tree
Plant vigour: Medium
Growth habit: Spreading
Canopy vegetation: Medium

Characters of the leaf
Shape: Lanceolate
Blade length: Long
Blade width: Medium

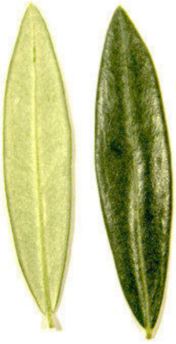

Characters of the inflorescence
Length: Short

Number of flowers for inflorescence: Low

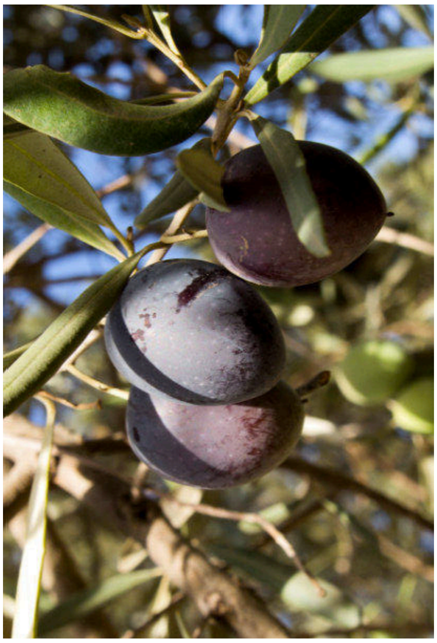

Characters of the fruit
Weight: Very high
Shape: Ovoid
Symmetry: Slightly asymmetric
Position of maximum transverse diameter: Towards base
Apex: Rounded
Base: Rounded
Nipple: Tenuous
Lenticels number: Few
Lenticels dimension: Large

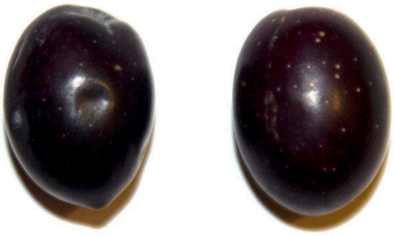

Characters of the endocarp
Weight: Very high
Shape: Elliptic
Symmetry: Slightly asymmetric
Position of maximum transverse diameter: Towards base
Apex: Pointed
Base: Rounded
Surface: Rugose
Number of grooves: High
Distribution of grooves: Uniform

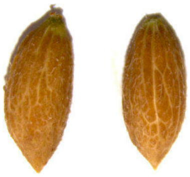

أيما ل
Aimel

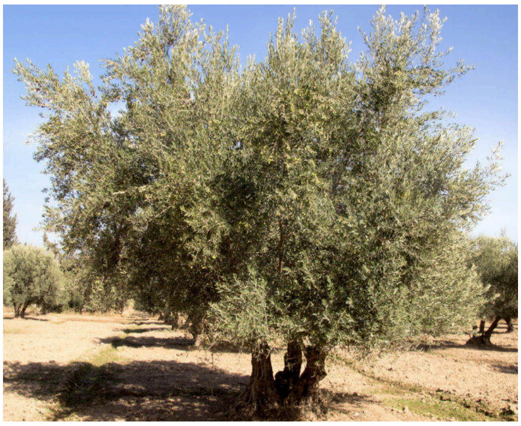

Characters of the tree
Plant vigour: Strong
Growth habit: Erect
Canopy vegetation: Medium

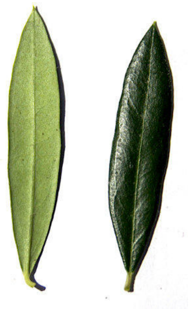

Characters of the leaf
Shape: Lanceolate
Blade length: Medium
Blade width: Medium

Characters of the inflorescence
Length: Short
Number of flowers for inflorescence: Low

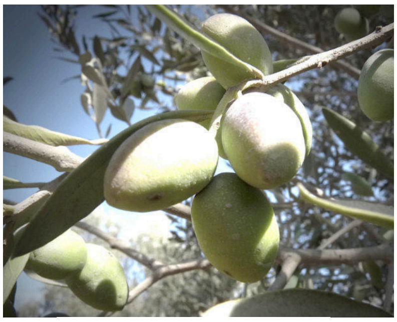

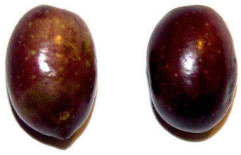

Characters of the fruit
Weight: Low
Shape: Elongated
Symmetry: Asymmetric
Position of maximum transverse diameter: Central
Apex: Pointed
Base: Truncate
Nipple: Absent
Lenticels number: Few
Lenticels dimension: Small

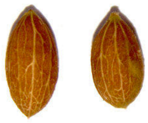

Characters of the endocarp
Weight: Medium
Shape: Elliptic
Symmetry: Slightly asymmetric
Position of maximum transverse diameter: Central apex
Apex: Rounded
Base: Rounded
Surface: Smooth
Number of grooves: Medium
Distribution of grooves: Uniform

أكرمة

Akerma

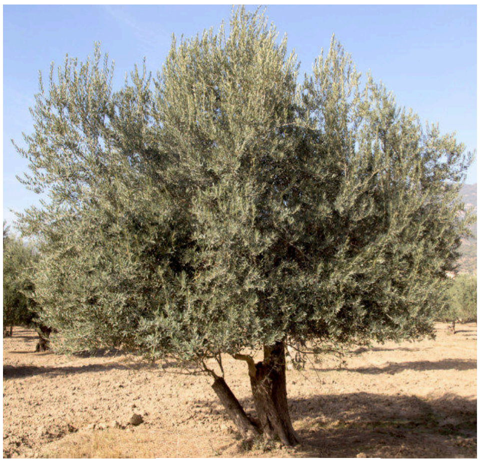

Characters of the tree
Plant vigour: Medium
Growth habit: Spreading
Canopy vegetation: Sparse

Characters of the leaf
Shape: Elliptic-Lanceolate
Blade length: Medium
Blade width: Medium

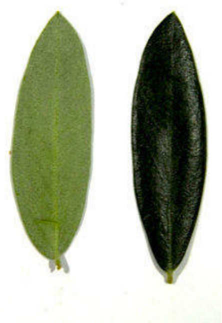

Characters of the inflorescence

Length: Short
Number of flowers for inflorescence: Low

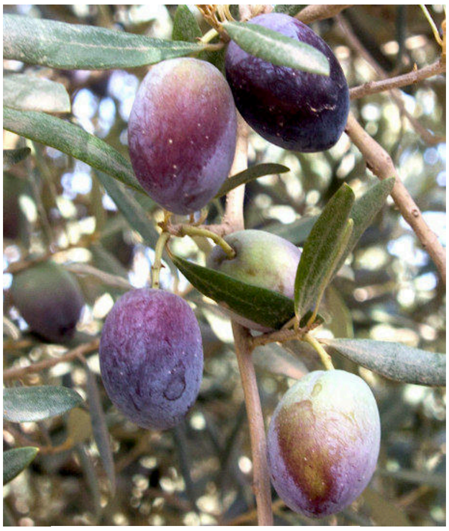

Characters of the fruit
Weight: High
Shape: Elongated
Symmetry: Slightly asymmetric
Position of maximum transverse diameter: Central
Apex: Pointed
Base: Rounded
Nipple: Absent
Lenticels number: Few
Lenticels dimension: Small

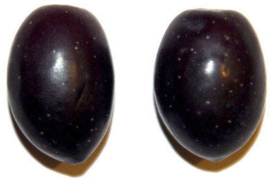

Characters of the endocarp
Weight: High
Shape: Elongated
Symmetry: Slightly asymmetric
Position of maximum transverse diameter: Central apex
Apex: Pointed
Base: Rounded
Surface: Rugose
Number of grooves: High
Distribution of grooves: Uniform

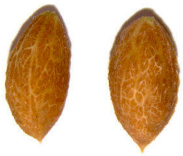

أزراج
Azeradj

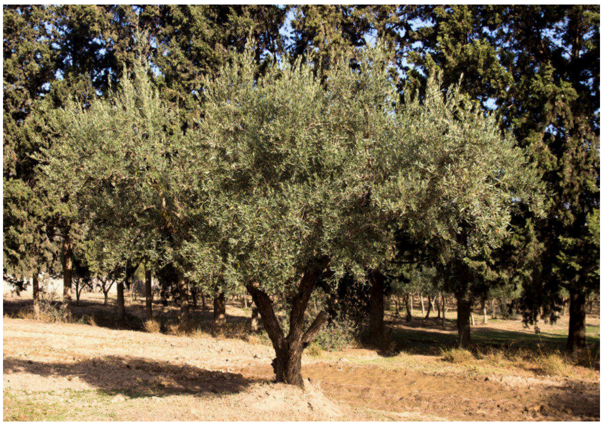

Characters of the tree
Plant vigour: Weak
Growth habit: Spreading
Canopy vegetation: Dense

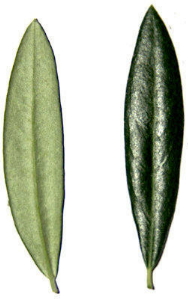

Characters of the leaf
Shape: Elliptic-Lanceolate
Blade length: Medium
Blade width: Medium

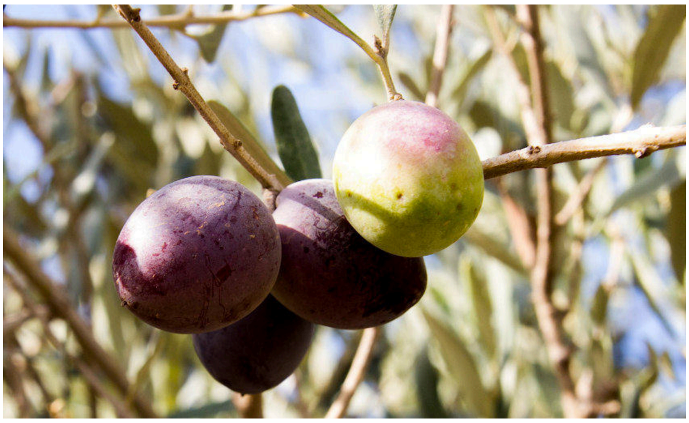

Characters of the inflorescence
Length: Short
Number of flowers for inflorescence: Low

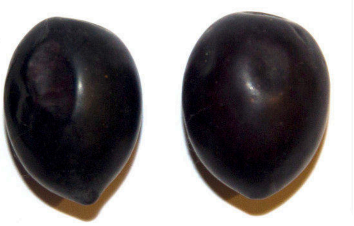

Characters of the fruit
Weight: High
Shape: Elongated
Symmetry: Slightly asymmetric
Position of maximum transverse diameter: Towards base
Apex: Pointed
Base: Rounded
Nipple: Tenuous
Lenticels number: Few
Lenticels dimension: Large

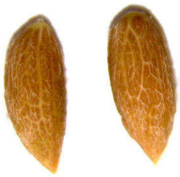

Characters of the endocarp
Weight: High
Shape: Elliptic
Symmetry: Slightly asymmetric
Position of maximum transverse diameter: Central apex
Apex: Pointed
Base: Rounded
Surface: Smooth
Number of grooves: Medium
Distribution of grooves: Uniform

بلانكات قالمة
Blanquette de Guelma

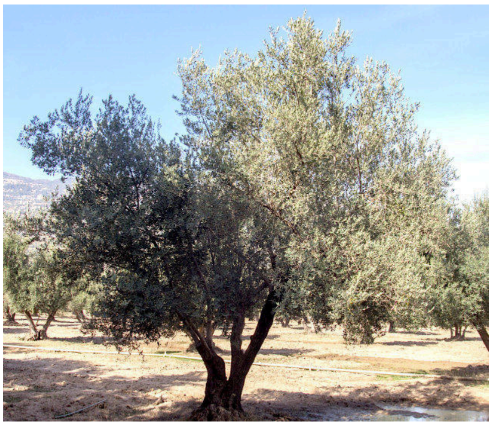

Characters of the tree
Plant vigour: Medium
Growth habit: Erect
Canopy vegetation: Sparse

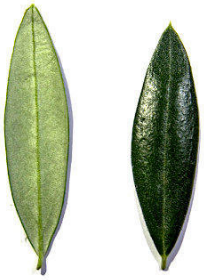

Characters of the leaf
Shape: Elliptic-Lanceolate
Blade length: Medium
Blade width: Medium

Characters of the inflorescence
Length: Medium
Number of flowers for inflorescence: Medium

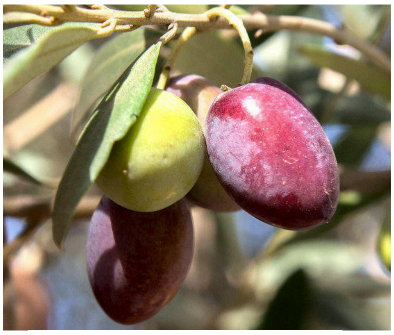

Characters of the fruit
Weight: Medium
Shape: Ovoid
Symmetry: Slightly asymmetric
Position of maximum transverse diameter: Towards base
Apex: Pointed
Base: Rounded
Nipple: Absent
Lenticels number: Many
Lenticels dimension: Small

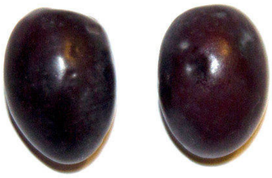

Characters of the endocarp
Weight: Medium
Shape: Elongated
Symmetry: Asymmetric
Position of maximum transverse diameter: Central apex
Apex: Pointed
Base: Rounded
Surface: Smooth
Number of grooves: Medium
Distribution of grooves: Uniform

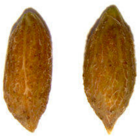

بو شوك قرقور
Bouchouk Guergour

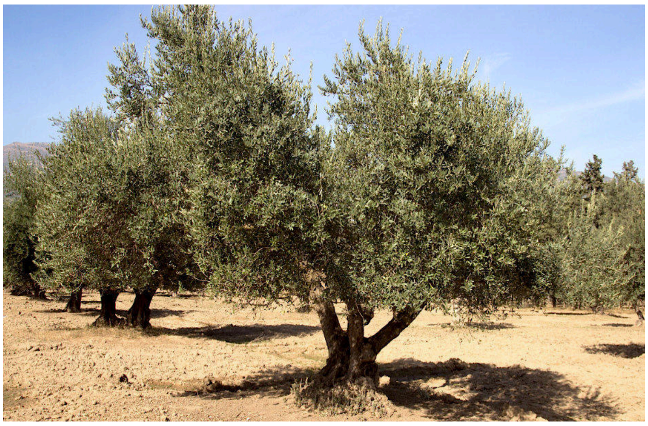

Characters of the tree
Plant vigour: Medium
Growth habit: Spreading
Canopy vegetation: Dense

Characters of the leaf
Shape: Elliptic-Lanceolate
Blade length: Medium
Blade width: Medium

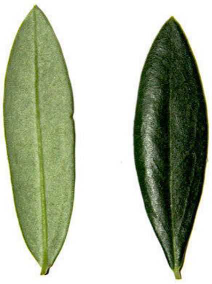

Characters of the inflorescence
Length: Short
Number of flowers for inflorescence: Low

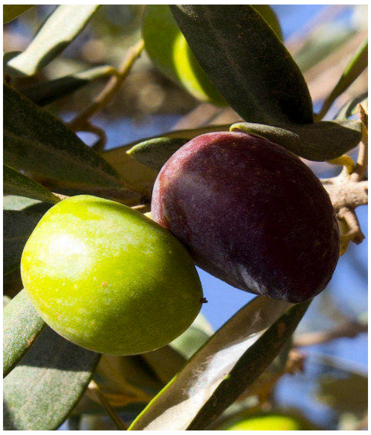

Characters of the fruit
Weight: High
Shape: Elongated
Symmetry: Asymmetric
Position of maximum transverse diameter: Towards base
Apex: Pointed
Base: Rounded
Nipple: Tenuous
Lenticels number: Few
Lenticels dimension: Large

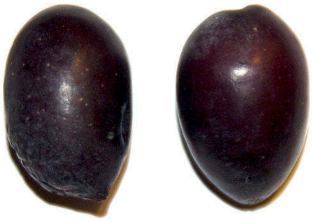

Characters of the endocarp
Weight: High
Shape: Elongated
Symmetry: Slightly asymmetric
Position of maximum transverse diameter: Central apex
Apex: Pointed
Base: Rounded
Surface: Rugose
Number of grooves: Medium
Distribution of grooves: Uniform

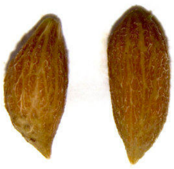

بو شوك لافاييت

Bouchouk Lafayette

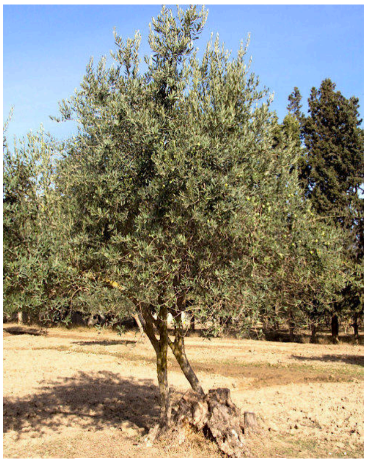

Characters of the tree
Plant vigour: Strong
Growth habit: Spreading
Canopy vegetation: Medium

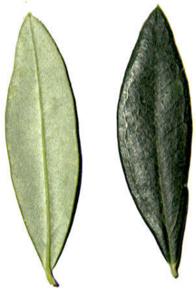

Characters of the leaf
Shape: Elliptic-Lanceolate
Blade length: Medium
Blade width: Medium

Characters of the inflorescence
Length: Short
Number of flowers for inflorescence: Medium

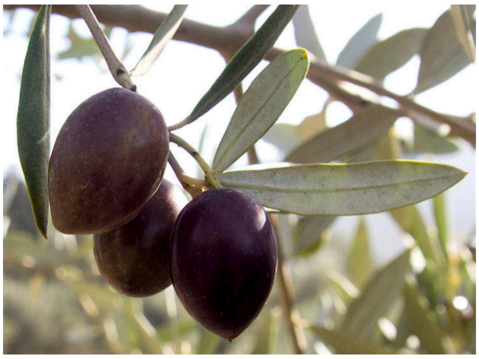

Characters of the fruit
Weight: Medium
Shape: Elongated
Symmetry: Asymmetric
Position of maximum transverse diameter: Towards base
Apex: Pointed
Base: Truncate
Nipple: Absent
Lenticels number: Few
Lenticels dimension: Large

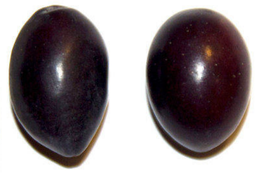

Characters of the endocarp
Weight: High
Shape: Elongated
Symmetry: Asymmetric
Position of maximum transverse diameter: Central apex
Apex: Pointed
Base: Pointed
Surface: Rugose
Number of grooves: Medium
Distribution of grooves: Uniform

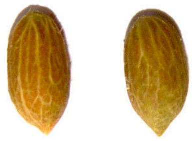

بوشوك صومام
Bouchouk Soummam

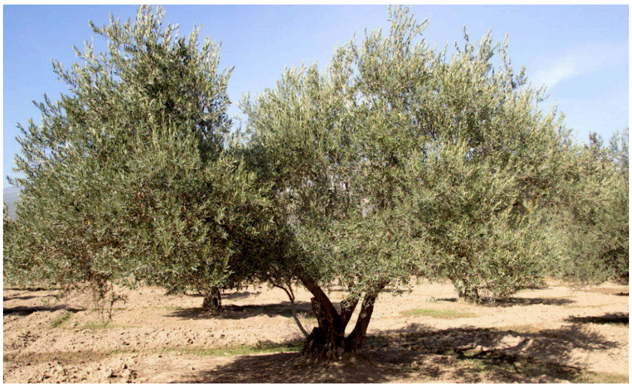

Characters of the tree
Plant vigour: Medium
Growth habit: Spreading
Canopy vegetation: Medium

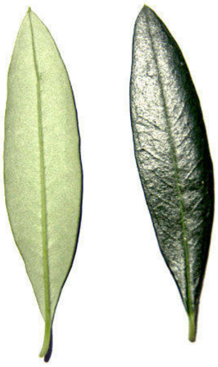

Characters of the leaf
Shape: Lanceolate
Blade length: Long
Blade width: Medium

Characters of the inflorescence
Length: Short
Number of flowers for inflorescence: Low

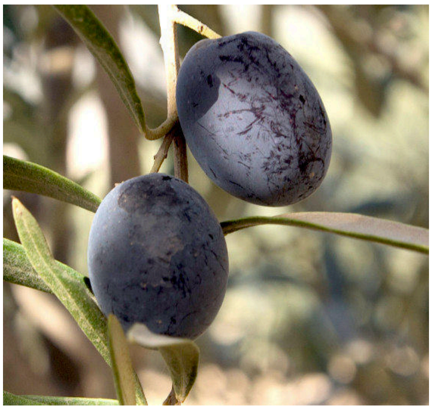

Characters of the fruit
Weight: High
Shape: Ovoid
Symmetry: Slightly asymmetric
Position of maximum transverse diameter: Central
Apex: Rounded
Base: Rounded
Nipple: Absent
Lenticels number: Few
Lenticels dimension: Large

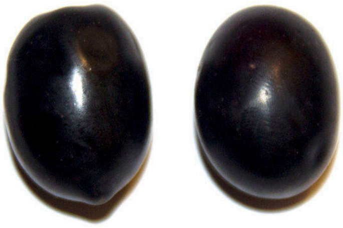

Characters of the endocarp
Weight: High
Shape: Elongated
Symmetry: Symmetric
Position of maximum transverse diameter: Towards apex
Apex: Rounded
Base: Rounded
Surface: Rugose
Number of grooves: High
Distribution of grooves: Uniform

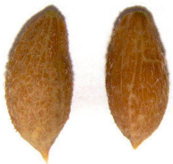

بوغنفوس
Boughenfous

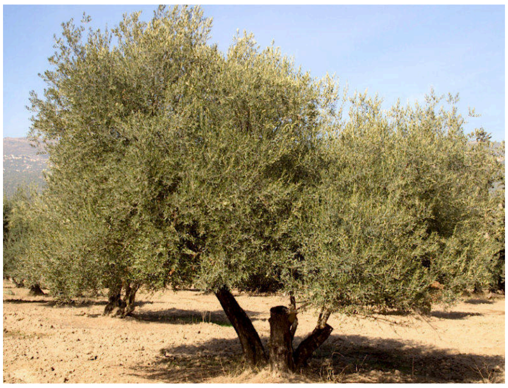

Characters of the tree
Plant vigour: Medium
Growth habit: Spreading
Canopy vegetation: Medium

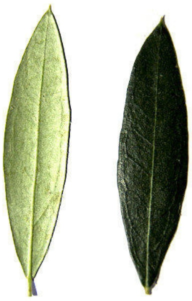

Characters of the leaf
Shape: Elliptic-Lanceolate
Blade length: Medium
Blade width: Medium

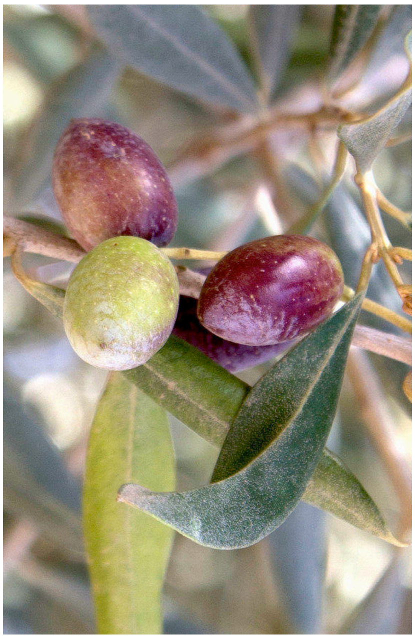

Characters of the inflorescence
Length: Medium
Number of flowers for inflorescence: High

Characters of the fruit
Weight: Low
Shape: Elongated
Symmetry: Slightly asymmetric
Position of maximum transverse diameter: Central
Apex: Pointed
Base: Truncate
Nipple: Absent
Lenticels number: Few
Lenticels dimension: Small

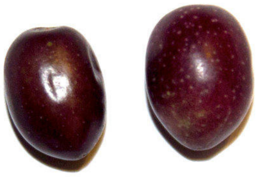

Characters of the endocarp
Weight: Low
Shape: Elliptic
Symmetry: Slightly asymmetric
Position of maximum transverse diameter: Central apex
Apex: Pointed
Base: Rounded
Surface: Smooth
Number of grooves: Medium
Distribution of grooves: Uniform

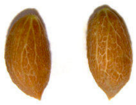

بويشرط
Bouichret

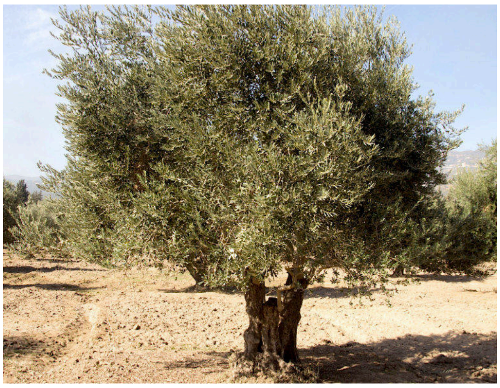

Characters of the tree
Plant vigour: Week
Growth habit: Erect
Canopy vegetation: Sparse

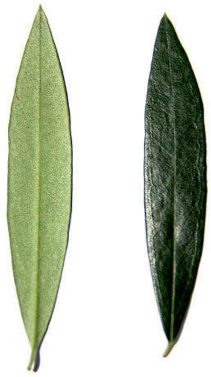

Characters of the leaf
Shape: Lanceolate
Blade length: Long
Blade width: Medium

Characters of the inflorescence
Length: Short
Number of flowers for inflorescence: Low

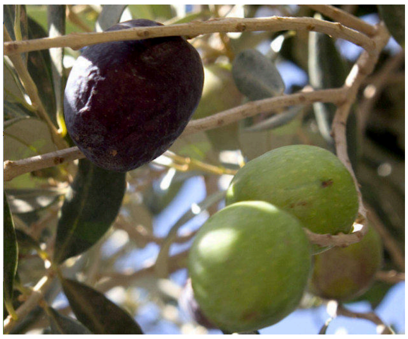

Characters of the fruit
Weight: Medium
Shape: Elongated
Symmetry: Asymmetric
Position of maximum transverse diameter: Towards base
Apex: Pointed
Base: Rounded
Nipple: Tenuous
Lenticels number: Few
Lenticels dimension: Large

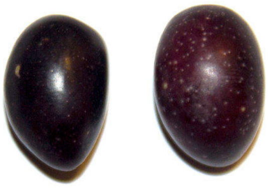

Characters of the endocarp
Weight: High
Shape: Elongated
Symmetry: Asymmetric
Position of maximum transverse diameter: Central apex
Apex: Pointed
Base: Rounded
Surface: Rugose
Number of grooves: Medium
Distribution of grooves: Uniform

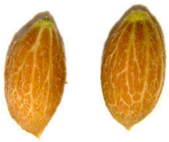

بوكيلة
Boukaila

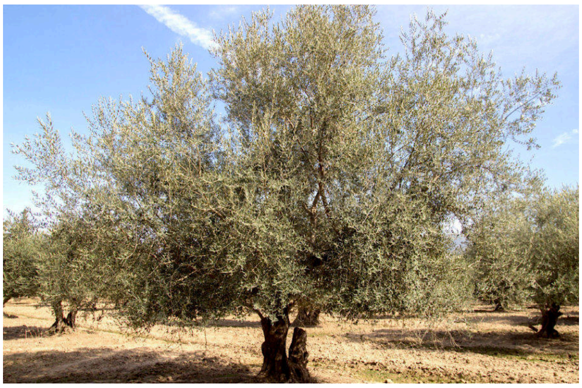

Characters of the tree
Plant vigour: Strong
Growth habit: Spreading
Canopy vegetation: Medium

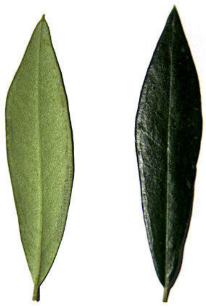

Characters of the leaf
Shape: Elliptic-Lanceolate
Blade length: Medium
Blade width: Medium

Characters of the inflorescence
Length: Medium
Number of flowers for inflorescence: Medium

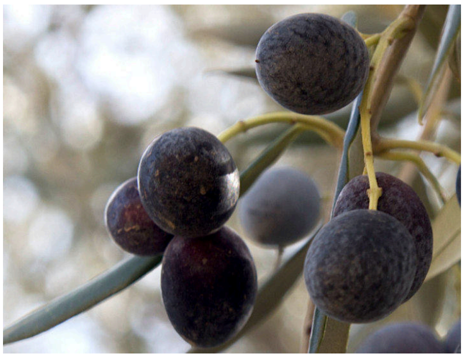

Characters of the fruit
Weight: Low
Shape: Ovoid
Symmetry: Slightly asymmetric
Position of maximum transverse diameter: Central
Apex: Pointed
Base: Rounded
Nipple: Absent
Lenticels number: Many
Lenticels dimension: Small

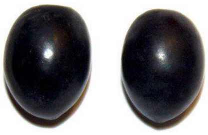

Characters of the endocarp
Weight: Low
Shape: Elliptic
Symmetry: Symmetric
Position of maximum transverse diameter: Central apex
Apex: Rounded
Base: Rounded
Surface: Smooth
Number of grooves: Medium
Distribution of grooves: Uniform

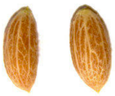

بوريشة
Bouricha

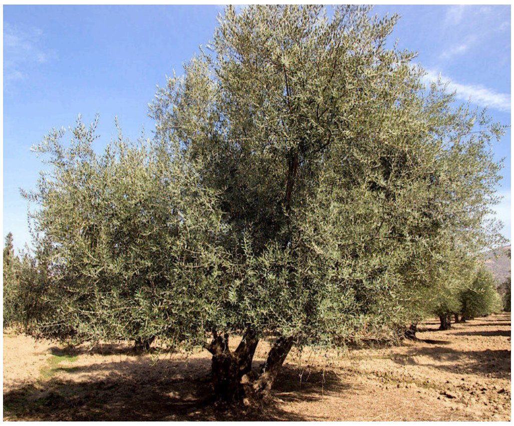

Characters of the tree
Plant vigour: Strong
Growth habit: Spreading
Canopy vegetation: Medium

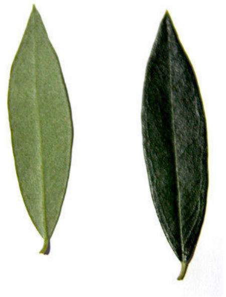

Characters of the leaf
Shape: Elliptic-Lanceolate
Blade length: Medium
Blade width: Medium

Characters of the inflorescence
Length: Medium
Number of flowers for inflorescence: Medium

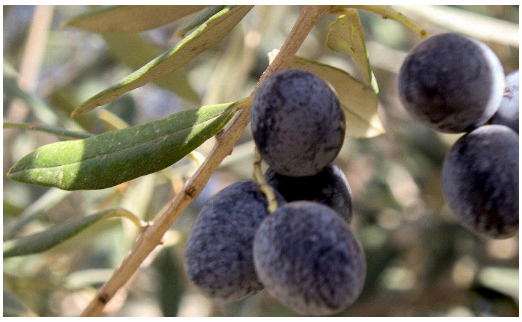

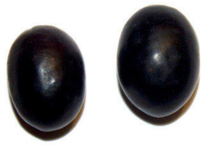

Characters of the fruit
Weight: Low
Shape: Elongated
Symmetry: Slightly asymmetric
Position of maximum transverse diameter: Central
Apex: Pointed
Base: Truncate
Nipple: Absent
Lenticels number: Few
Lenticels dimension: Small

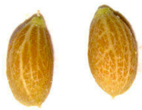

Characters of the endocarp
Weight: Medium
Shape: Elongated
Symmetry: Slightly asymmetric
Position of maximum transverse diameter: Central apex
Apex: Pointed
Base: Pointed
Surface: Smooth
Number of grooves: Medium
Distribution of grooves: Uniform

شملال
Chemlal

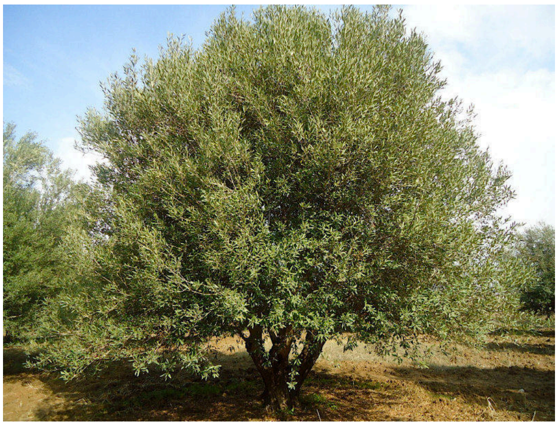

Characters of the tree
Plant vigour: Strong
Growth habit: Erect
Canopy vegetation: Medium

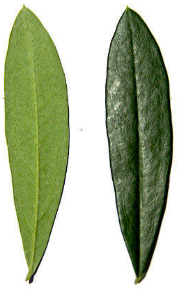

Characters of the leaf
Shape: Elliptic-Lanceolate
Blade length: Medium
Blade width: Medium

Characters of the inflorescence
Length: Medium
Number of flowers for inflorescence: Medium

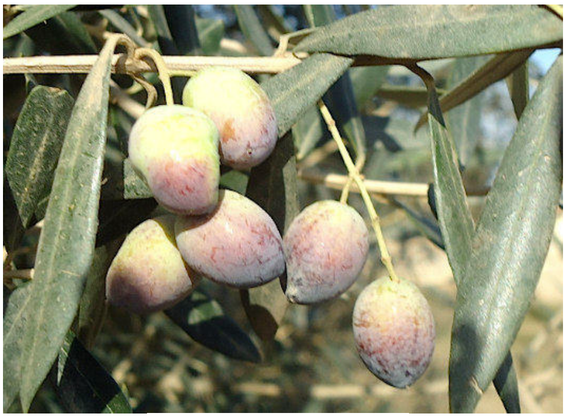

Characters of the fruit
Weight: Low
Shape: Elongated
Symmetry: Asymmetric
Position of maximum transverse diameter: Central
Apex: Pointed
Base: Rounded
Nipple: Absent
Lenticels number: Many
Lenticels dimension: Small

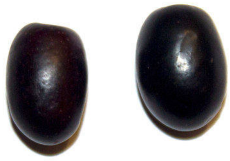

Characters of the endocarp
Weight: Medium
Shape: Elliptic
Symmetry: Slightly asymmetric
Position of maximum transverse diameter: Central apex
Apex: Pointed
Base: Rounded
Surface: Smooth
Number of grooves: Medium
Distribution of grooves: Uniform

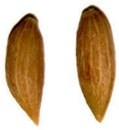

فركاني
Ferkani

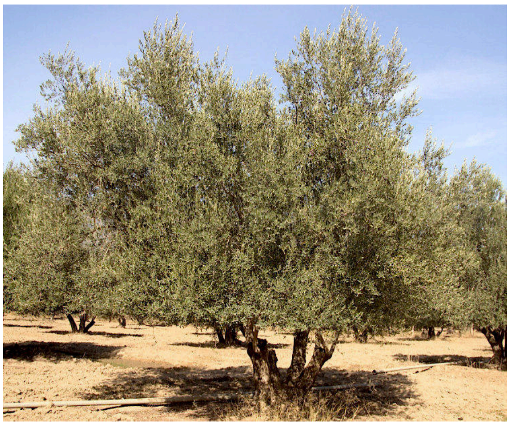

Characters of the tree
Plant vigour: Medium
Growth habit: Spreading
Canopy vegetation: Sparse

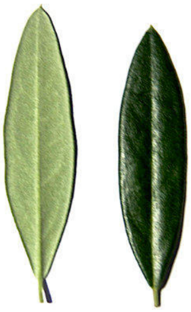

Characters of the leaf
Shape: Elliptic-Lanceolate
Blade length: Medium
Blade width: Medium

Characters of the inflorescence
Length: Medium
Number of flowers for inflorescence: Low

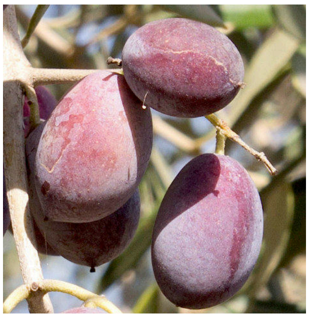

Characters of the fruit
Weight: Medium
Shape: Elongated
Symmetry: Slightly asymmetric
Position of maximum transverse diameter: Central
Apex: Rounded
Base: Rounded
Nipple: Obvious
Lenticels number: Few
Lenticels dimension: Small

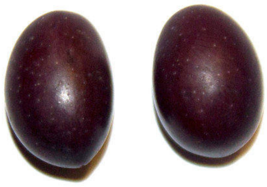

Characters of the endocarp
Weight: Medium
Shape: Elongated
Symmetry: Slightly asymmetric
Position of maximum transverse diameter: Central apex
Apex: Pointed
Base: Pointed
Surface: Smooth
Number of grooves: Medium
Distribution of grooves: Uniform

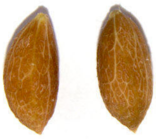

كبيرة الحامة

Grosse du Hamma

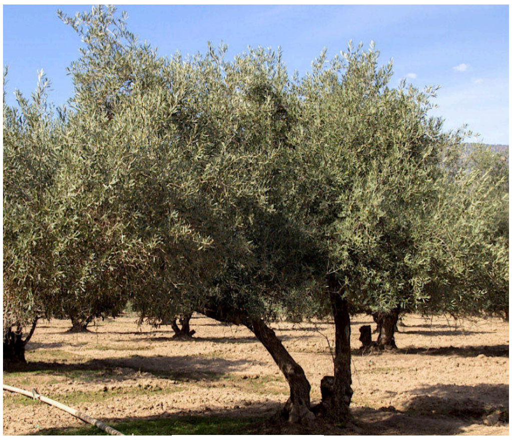

Characters of the tree
Plant vigour: Medium
Growth habit: Spreading
Canopy vegetation: Dense

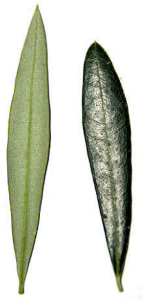

Characters of the leaf
Shape: Lanceolate
Blade length: Medium
Blade width: Medium

Characters of the inflorescence
Length: Short
Number of flowers for inflorescence: Low

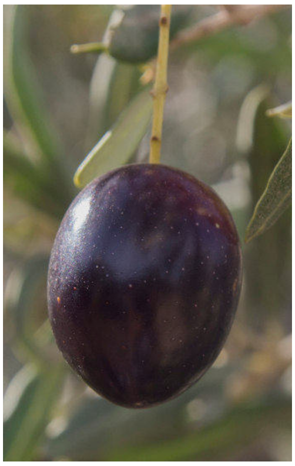

Characters of the fruit
Weight: Very high
Shape: Elongated
Symmetry: Asymmetric
Position of maximum transverse diameter: Towards base
Apex: Rounded
Base: Rounded
Nipple: Tenuous
Lenticels number: Few
Lenticels dimension: Large

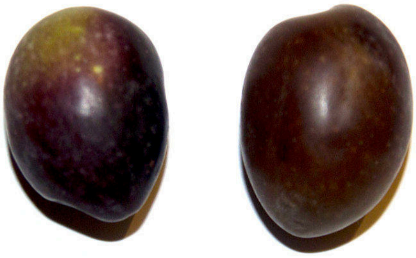

Characters of the endocarp
Weight: Very high
Shape: Elongated
Symmetry: Asymmetric
Position of maximum transverse diameter: Towards base
Apex: Pointed
Base: Rounded
Surface: Scabrous
Number of grooves: Medium
Distribution of grooves: Uniform

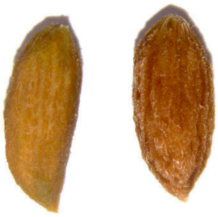

حمراء
Hamra

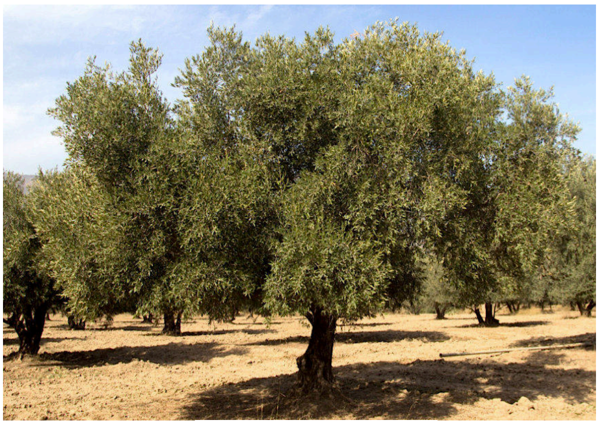

Characters of the tree
Plant vigour: Strong
Growth habit: Erect
Canopy vegetation: Medium

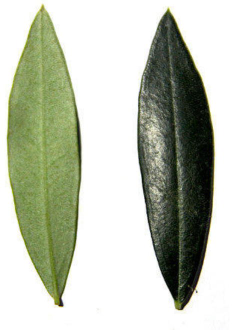

Characters of the leaf
Shape: Elliptic-Lanceolate
Blade length: Medium
Blade width: Medium

Characters of the inflorescence
Length: Medium
Number of flowers for inflorescence: Medium

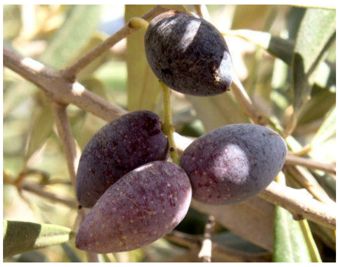

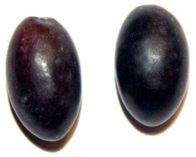

Characters of the fruit
Weight: Low
Shape: Ovoid
Symmetry: Slightly asymmetric
Position of maximum transverse diameter: Central
Apex: Rounded
Base: Rounded
Nipple: Absent
Lenticels number: Many
Lenticels dimension: Small

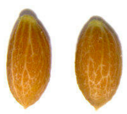

Characters of the endocarp
Weight: Medium
Shape: Elliptic
Symmetry: Slightly asymmetric
Position of maximum transverse diameter: Central apex
Apex: Rounded
Base: Rounded
Surface: Smooth
Number of grooves: Medium
Distribution of grooves: Uniform

ليملي
Limli

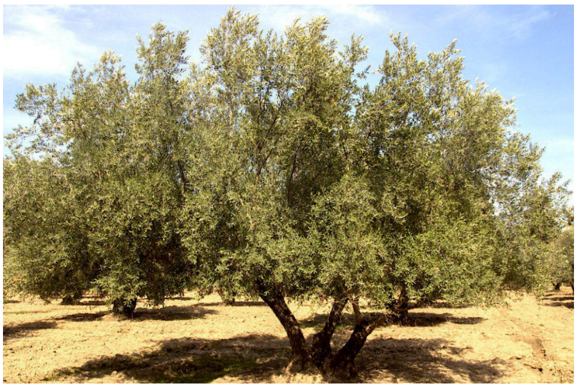

Characters of the tree
Plant vigour: Medium
Growth habit: Spreading
Canopy vegetation: Medium

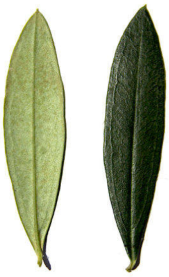

Characters of the leaf
Shape: Elliptic-Lanceolate
Blade length: Medium
Blade width: Medium

Characters of the inflorescence
Length: Medium
Number of flowers for inflorescence: Medium

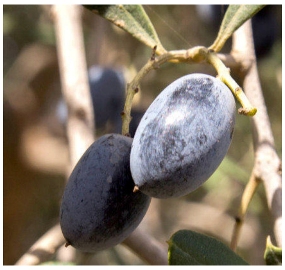

Characters of the fruit
Weight: Low
Shape: Elongated
Symmetry: Slightly asymmetric
Position of maximum transverse diameter: Central
Apex: Pointed
Base: Truncate
Nipple: Absent
Lenticels number: Few
Lenticels dimension: Small

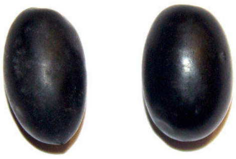

Characters of the endocarp
Weight: Medium
Shape: Elongated
Symmetry: Slightly asymmetric
Position of maximum transverse diameter: Central apex
Apex: Pointed
Base: Pointed
Surface: Smooth
Number of grooves: Medium
Distribution of grooves: Uniform

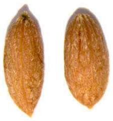

طويلة مليانة
Longue de Miliana

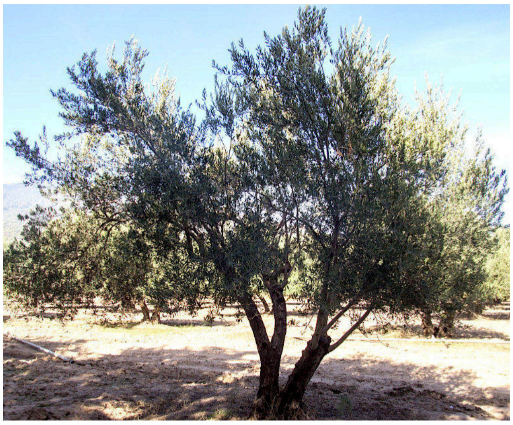

Characters of the tree
Plant vigour: Medium
Growth habit: Spreading
Canopy vegetation: Medium

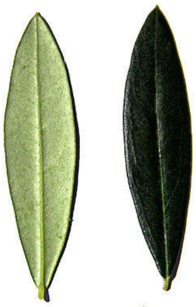

Characters of the leaf
Shape: Elliptic-Lanceolate
Blade length: Medium
Blade width: Medium

Characters of the inflorescence
Length: Short
Number of flowers for inflorescence: Medium

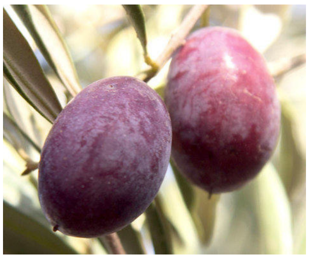

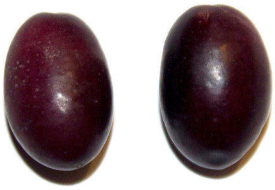

Characters of the fruit
Weight: Medium
Shape: Spherical
Symmetry: Slightly asymmetric
Position of maximum transverse diameter: Central
Apex: Rounded
Base: Rounded
Nipple: Absent
Lenticels number: Few
Lenticels dimension: Large

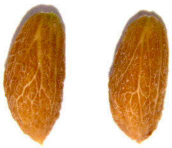

Characters of the endocarp
Weight: High
Shape: Ovoid
Symmetry: Slightly asymmetric
Position of maximum transverse diameter: Central apex
Apex: Rounded
Base: Rounded
Surface: Scabrous
Number of grooves: Medium
Distribution of grooves: Uniform

مكي
Mekki

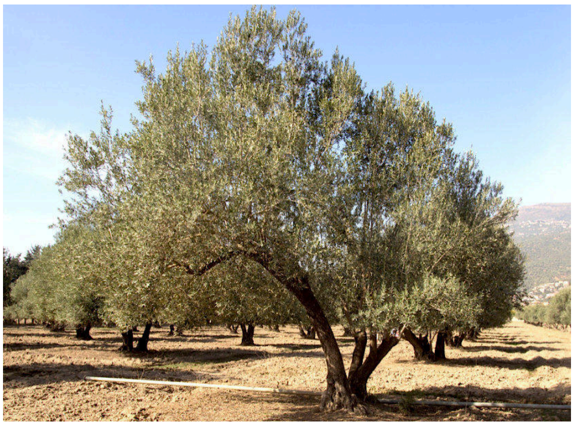

Characters of the tree
Plant vigour: Medium
Growth habit: Spreading
Canopy vegetation: Medium

Characters of the leaf
Shape: Elliptic-Lanceolate
Blade length: Medium
Blade width: Medium

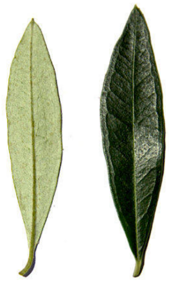

Characters of the inflorescence
Length: Short
Number of flowers for inflorescence: Low

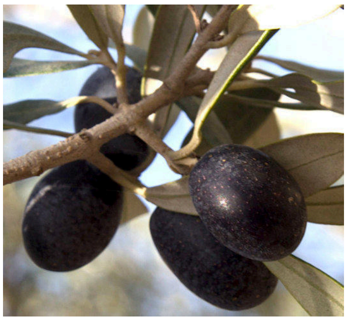

Characters of the fruit
Weight: Low
Shape: Ovoid
Symmetry: Slightly asymmetric
Position of maximum transverse diameter: Central
Apex: Rounded
Base: Truncate
Nipple: Absent
Lenticels number: Many
Lenticels dimension: Small

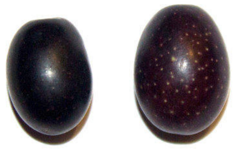

Characters of the endocarp
Weight: Medium
Shape: Elliptic
Symmetry: Slightly asymmetric
Position of maximum transverse diameter: Towards apex
Apex: Pointed
Base: Rounded
Surface: Smooth
Number of grooves: Medium
Distribution of grooves: Uniform

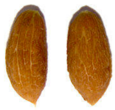

ناب الجمل

Neb Djemel

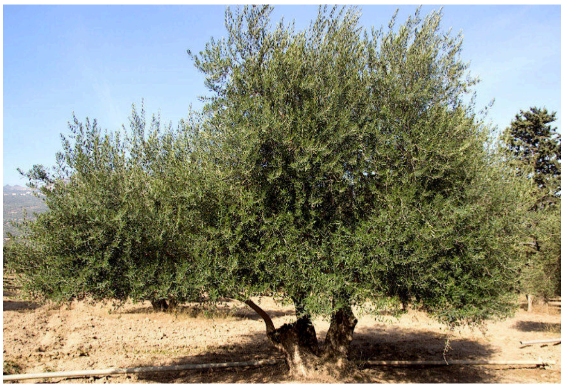

Characters of the tree
Plant vigour: Strong
Growth habit: Spreading
Canopy vegetation: Medium

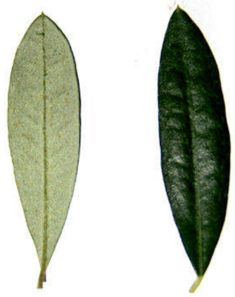

Characters of the leaf
Shape: Elliptic-Lanceolate
Blade length: Medium
Blade width: Medium

Characters of the inflorescence
Length: Medium
Number of flowers for inflorescence: Medium

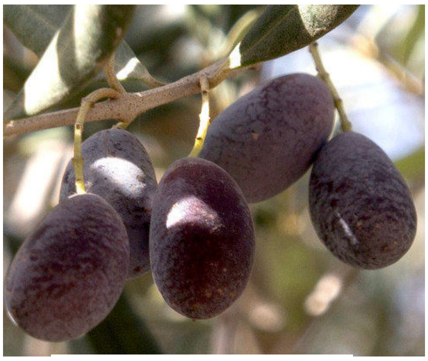

Characters of the fruit
Weight: Medium
Shape: Elongated
Symmetry: Asymmetric
Position of maximum transverse diameter: Central
Apex: Pointed
Base: Truncate
Nipple: Absent
Lenticels number: Many
Lenticels dimension: Small

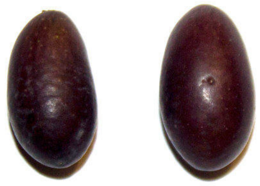

Characters of the endocarp
Weight: High
Shape: Elongated
Symmetry: Asymmetric
Position of maximum transverse diameter: Towards apex
Apex: Pointed
Base: Pointed
Surface: Smooth
Number of grooves: Medium
Distribution of grooves: Uniform

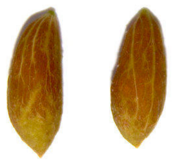

مستديرة مليانة

Ronde de Miliana

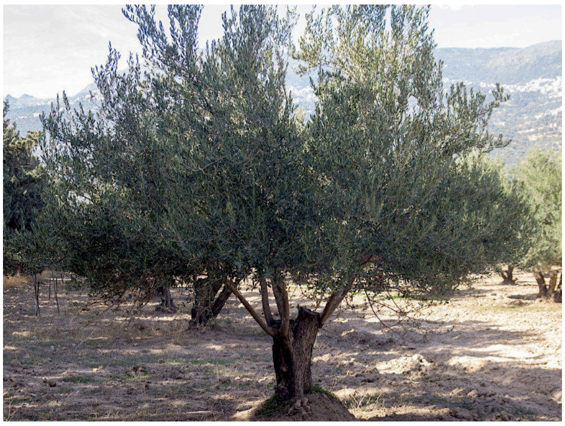

Characters of the tree
Plant vigour: Weak
Growth habit: Spreading
Canopy vegetation: Medium

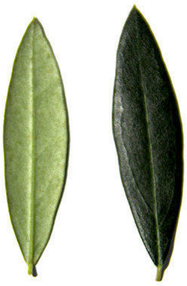

Characters of the leaf
Shape: Elliptic-Lanceolate
Blade length: Medium
Blade width: Medium

Characters of the inflorescence
Length: Medium
Number of flowers for inflorescence: Medium

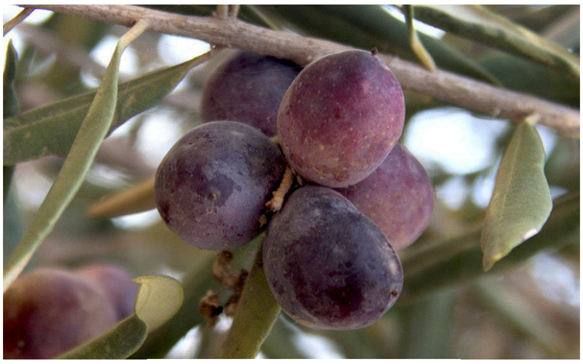

Characters of the fruit
Weight: Medium
Shape: Elongated
Symmetry: Slightly asymmetric
Position of maximum transverse diameter: Central
Apex: Rounded
Base: Rounded
Nipple: Absent
Lenticels number: Few
Lenticels dimension: Large

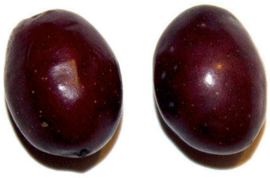

Characters of the endocarp
Weight: Very high
Shape: Elliptic
Symmetry: Slightly asymmetric
Position of maximum transverse diameter: Central apex
Apex: Pointed
Base: Rounded
Surface: Scabrous
Number of grooves: Medium
Distribution of grooves: Uniform

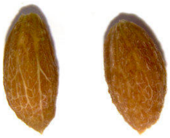

حمراء متيجة
Rougette de Mitidja

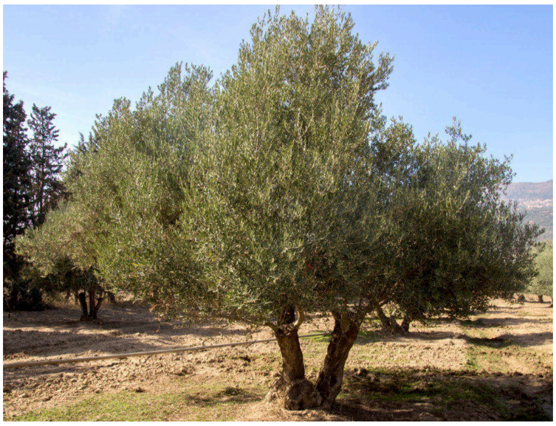

Characters of the tree
Plant vigour: Medium
Growth habit: Spreading
Canopy vegetation: Medium

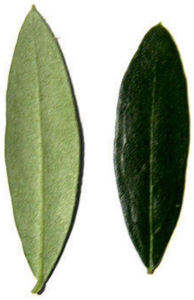

Characters of the leaf
Shape: Elliptic-Lanceolate
Blade length: Medium
Blade width: Medium

Characters of the inflorescence
Length: Medium
Number of flowers for inflorescence: Low

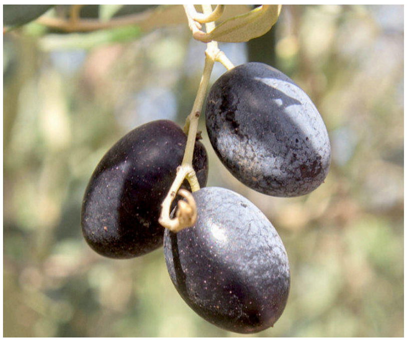

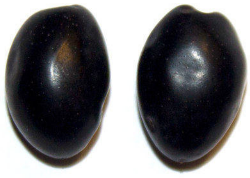

Characters of the fruit
Weight: Medium
Shape: Elongated
Symmetry: Slightly asymmetric
Position of maximum transverse diameter: Central
Apex: Pointed
Base: Truncate
Nipple: Absent
Lenticels number: Few
Lenticels dimension: Small

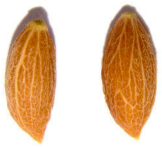

Characters of the endocarp
Weight: High
Shape: Elongated
Symmetry: Asymmetric
Position of maximum transverse diameter: Central apex
Apex: Pointed
Base: Pointed
Surface: Smooth
Number of grooves: Medium
Distribution of grooves: Uniform

سجواز
Sigoise

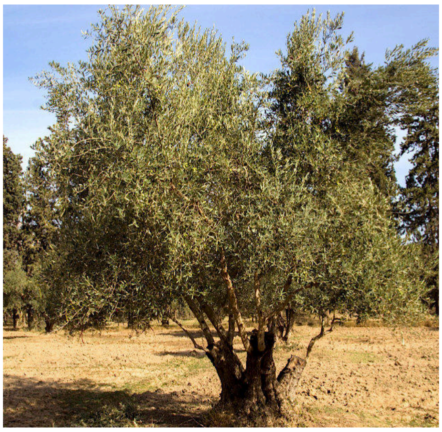

Characters of the tree
Plant vigour: Medium
Growth habit: Erect
Canopy vegetation: Medium

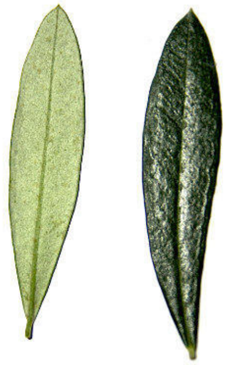

Characters of the leaf
Shape: Elliptic-Lanceolate
Blade length: Long
Blade width: Medium

Characters of the inflorescence
Length: Medium
Number of flowers for inflorescence: Low

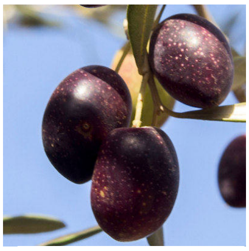

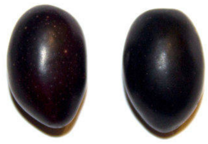

Characters of the fruit
Weight: Low
Shape: Ovoid
Symmetry: Slightly asymmetric
Position of maximum transverse diameter: Central
Apex: Pointed
Base: Truncate
Nipple: Absent
Lenticels number: Many
Lenticels dimension: Small

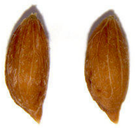

Characters of the endocarp
Weight: Medium
Shape: Elliptic
Symmetry: Asymmetric
Position of maximum transverse diameter: Central apex
Apex: Pointed
Base: Rounded
Surface: Smooth
Number of grooves: Medium
Distribution of grooves: Uniform

سويدي
Souidi

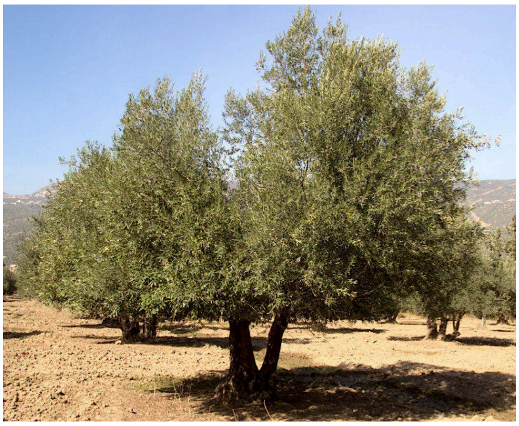

Characters of the tree
Plant vigour: Medium
Growth habit: Erect
Canopy vegetation: Sparse

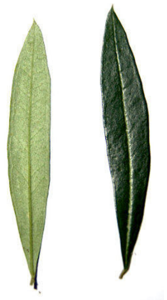

Characters of the leaf
Shape: Lanceolate
Blade length: Medium
Blade width: Medium

Characters of the inflorescence
Length: Medium
Number of flowers for inflorescence: Medium

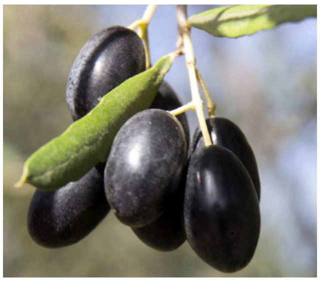

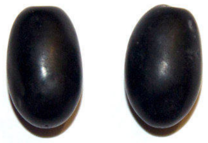

Characters of the fruit
Weight: Low
Shape: Elongated
Symmetry: Slightly asymmetric
Position of maximum transverse diameter: Central
Apex: Rounded
Base: Truncate
Nipple: Absent
Lenticels number: Few
Lenticels dimension: Small

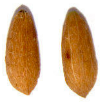

Characters of the endocarp
Weight: Low
Shape: Elongated
Symmetry: Slightly asymmetric
Position of maximum transverse diameter: Towards apex
Apex: Rounded
Base: Pointed
Surface: Smooth
Number of grooves: Medium
Distribution of grooves: Uniform

تبلوط
Tabelout

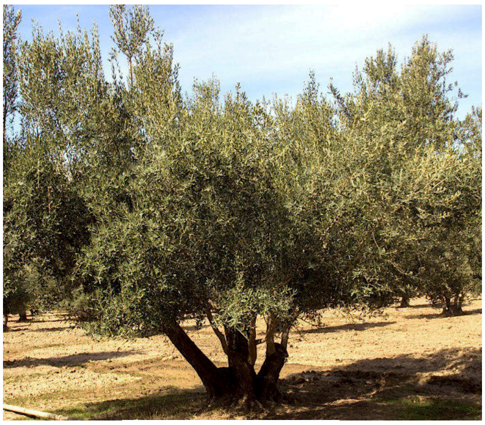

Characters of the tree
Plant vigour: Strong
Growth habit: Spreading
Canopy vegetation: Medium

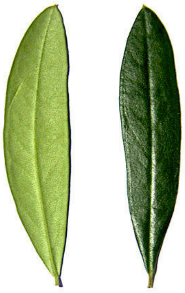

Characters of the leaf
Shape: Elliptic-Lanceolate
Blade length: Medium
Blade width: Medium

Characters of the inflorescence
Length: Medium
Number of flowers for inflorescence: High

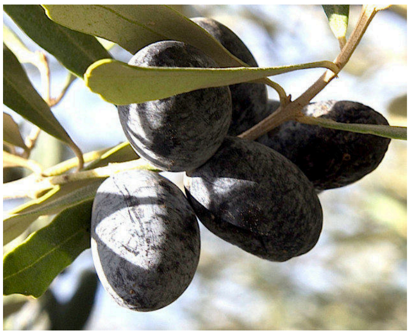

Characters of the fruit
Weight: Medium
Shape: Elongated
Symmetry: Slightly asymmetric
Position of maximum transverse diameter: Central
Apex: Pointed
Base: Truncate
Nipple: Absent
Lenticels number: Few
Lenticels dimension: Small

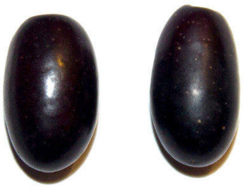

Characters of the endocarp
Weight: High
Shape: Elongated
Symmetry: Asymmetric
Position of maximum transverse diameter: Central apex
Apex: Pointed
Base: Pointed
Surface: Rugose
Number of grooves: Medium
Distribution of grooves: Uniform

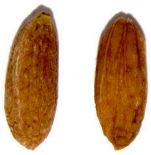

تكسريت

Takesrit

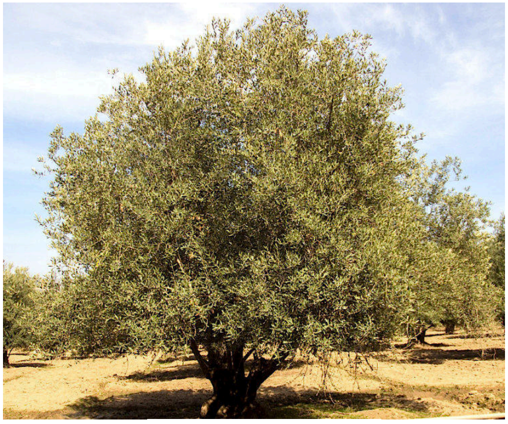

Characters of the tree
Plant vigour: Medium
Growth habit: Spreading
Canopy vegetation: Medium

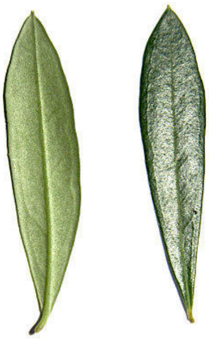

Characters of the leaf
Shape: Elliptic-Lanceolate
Blade length: Medium
Blade width: Medium

Characters of the inflorescence
Length: Medium
Number of flowers for inflorescence: Medium

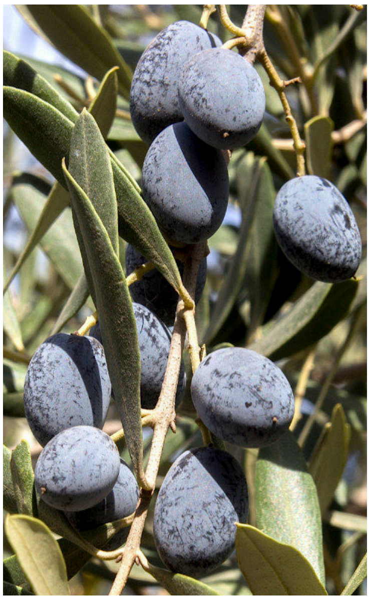

Characters of the fruit
Weight: Low
Shape: Elongated
Symmetry: Slightly asymmetric
Position of maximum transverse diameter: Central
Apex: Pointed
Base: Truncate
Nipple: Absent
Lenticels number: Few
Lenticels dimension: Small

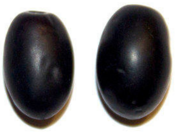

Characters of the endocarp
Weight: Medium
Shape: Elongated
Symmetry: Asymmetric
Position of maximum transverse diameter: Central apex
Apex: pointed
Base: Pointed
Surface: Smooth
Number of grooves: Medium
Distribution of grooves: Uniform

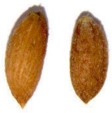

تفاح
Tefah

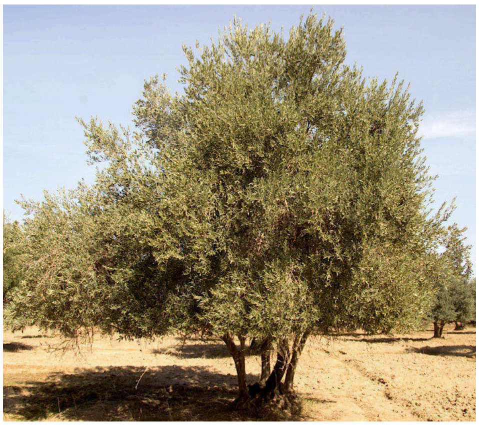

Characters of the tree
Plant vigour: Strong
Growth habit: Erect
Canopy vegetation: Medium

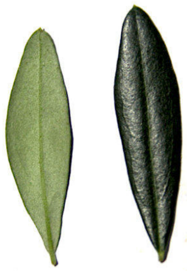

Characters of the leaf
Shape: Elliptic-Lanceolate
Blade length: Medium
Blade width: Medium

Characters of the inflorescence
Length: Short
Number of flowers for inflorescence: Low

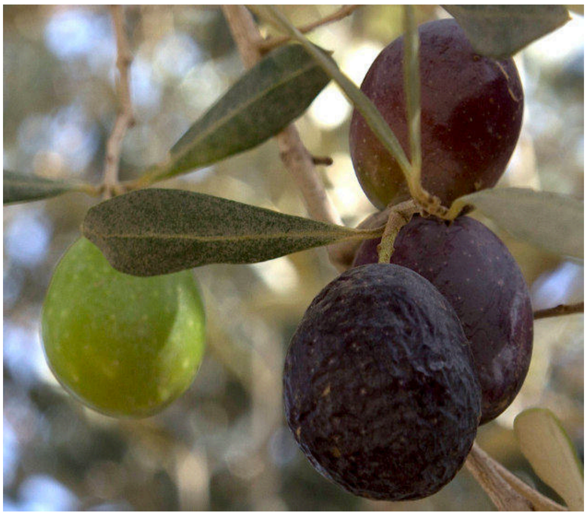

Characters of the fruit
Weight: Very high
Shape: Spherical
Symmetry: Slightly asymmetric
Position of maximum transverse diameter: Central
Apex: Rounded
Base: Rounded
Nipple: Absent
Lenticels number: Many
Lenticels dimension: Small

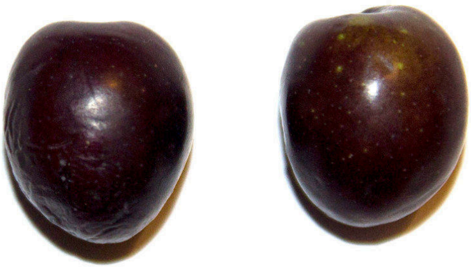

Characters of the endocarp
Weight: High
Shape: Ovoid
Symmetry: Slightly asymmetric
Position of maximum transverse diameter: Central apex
Apex: Rounded
Base: Rounded
Surface: Rugose
Number of grooves: Medium
Distribution of grooves: Uniform

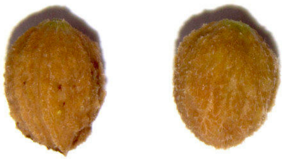

Supplement: Supplementary file 1 [file genes-11-00303-s001.zip › 12_Table_S2.pdf]
