# Supplementary figures and images for: Molecular Analysis of the Official Algerian Olive Collection Highlighted a Hotspot of Biodiversity in the Central Mediterranean Basin
Source: Genes (Basel). 2020 Mar 13;11(3):303. doi: 10.3390/genes11030303 (PMC7140851; doi:10.3390/genes11030303)

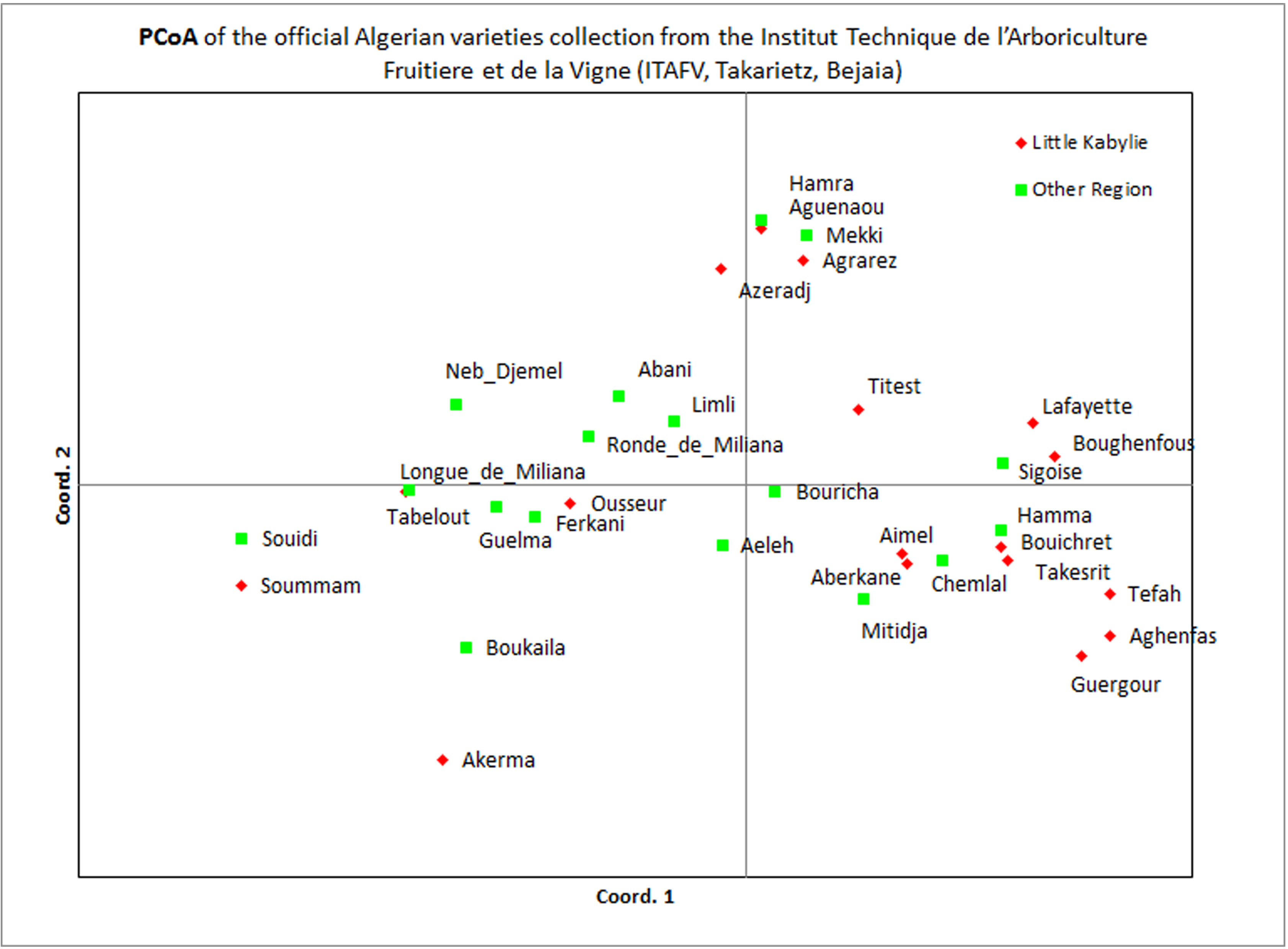

Supplement: Supplementary file 1 [file genes-11-00303-s001.zip › 9_Figure_S1.tif]

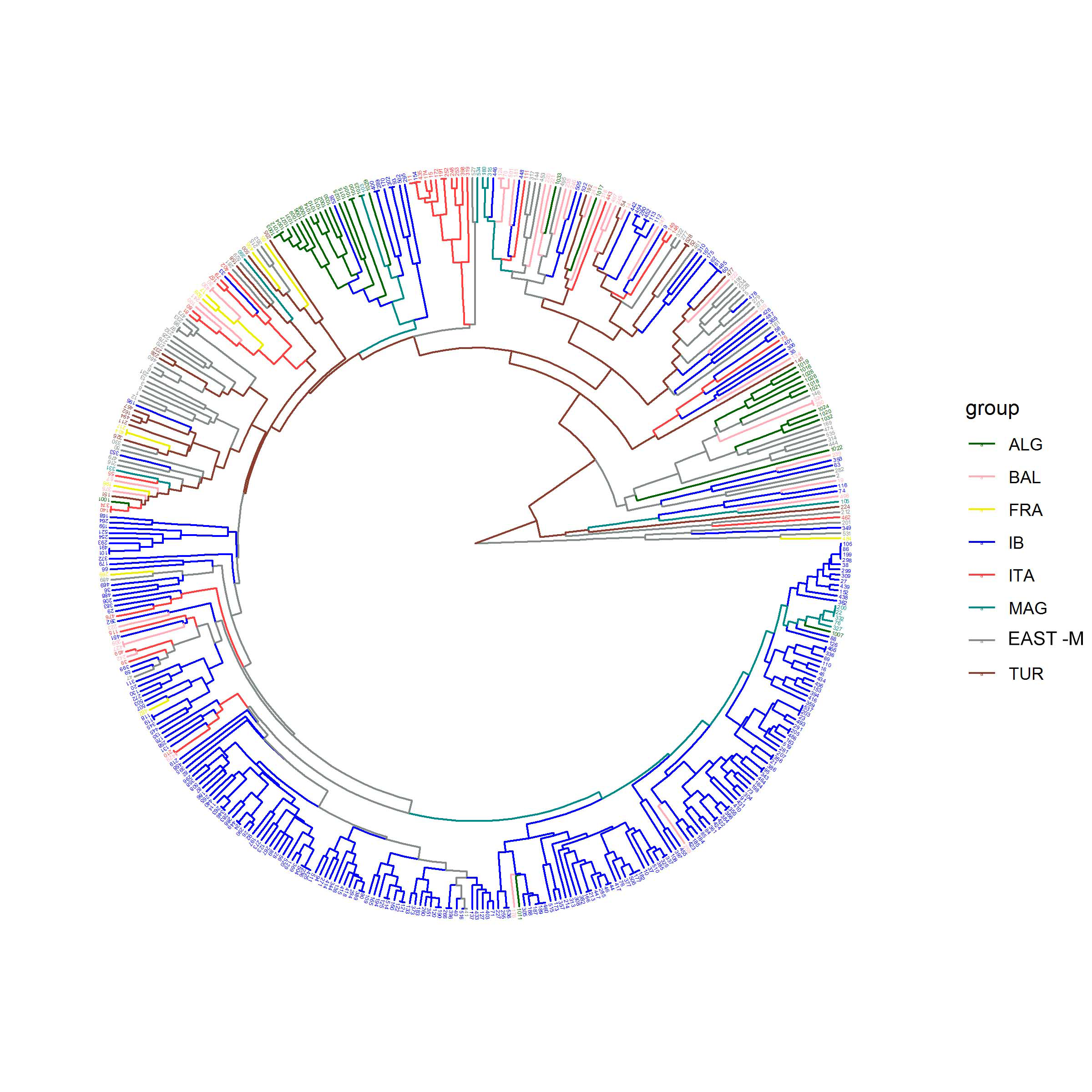

Supplement: Supplementary file 1 [file genes-11-00303-s001.zip › 10_Figure_S2.tif]
